# Tricyclic antipsychotics and antidepressants can inhibit α5‐containing GABA_A_ receptors by two distinct mechanisms

**DOI:** 10.1111/bph.15807

**Published:** 2022-03-07

**Authors:** Konstantina Bampali, Filip Koniuszewski, Luca L. Silva, Sabah Rehman, Florian D. Vogel, Thomas Seidel, Petra Scholze, Florian Zirpel, Arthur Garon, Thierry Langer, Matthäus Willeit, Margot Ernst

**Affiliations:** ^1^ Department of Pathobiology of the Nervous System, Center for Brain Research Medical University Vienna Vienna Austria; ^2^ Department of Molecular Neurosciences, Center for Brain Research Medical University of Vienna Vienna Austria; ^3^ Department of Pharmaceutical Sciences, Division of Pharmaceutical Chemistry University of Vienna Vienna Austria; ^4^ Department of Psychiatry and Psychotherapy Medical University of Vienna Vienna Austria

**Keywords:** allosteric modulation, antipsychotics, chlorpromazine, clozapine, functional inhibition, GABA_A_ receptor

## Abstract

**Background and Purpose:**

Many psychotherapeutic drugs, including clozapine, display polypharmacology and act on GABA_A_ receptors. Patients with schizophrenia show alterations in function, structure and molecular composition of the hippocampus, and a recent study demonstrated aberrant levels of hippocampal α5 subunit‐containing GABA_A_ receptors. The purpose of this study is to investigate the effects of tricyclic compounds on α5 subunit‐containing receptor subtypes.

**Experimental Approach:**

Functional studies of effects by seven antipsychotic and antidepressant medications were performed in several GABA_A_ receptor subtypes by two‐electrode voltage‐clamp electrophysiology using *Xenopus laevis* oocytes. Computational structural analysis was employed to design mutated constructs of the α5 subunit, probing a novel binding site. Radioligand displacement data complemented the functional and mutational findings.

**Key Results:**

The antipsychotic drugs clozapine and chlorpromazine exerted functional inhibition on multiple GABA_A_ receptor subtypes, including those containing α5‐subunits. Based on a chlorpromazine binding site observed in a GABA‐gated bacterial homologue, we identified a novel site in α5 GABA_A_ receptor subunits and demonstrate differential usage of this and the orthosteric sites by these ligands.

**Conclusion and Implications:**

Despite high molecular and functional similarities among the tested ligands, they reduce GABA currents by differential usage of allosteric and orthosteric sites. The chlorpromazine site we describe here is a new potential target for optimizing antipsychotic medications with beneficial polypharmacology. Further studies in defined subtypes are needed to substantiate mechanistic links between the therapeutic effects of clozapine and its action on certain GABA_A_ receptor subtypes.

**Abbreviations**
ECD: extracellular domain
ELIC: *Erwinia* ligand‐gated ion channel
NAM: negative allosteric modulation
PDB: Protein Data Bank
PET: positron emission tomography
TBPS: [35*S*]*t*‐butylbicyclophosphorothionate
TEVC: two‐electrode voltage clamp
THDOC: tetrahydrodeoxycorticosterone

**What is already known**

- Clozapine and other tricyclic molecules reduce GABA effects at ionotropic GABA receptors.
- Chlorpromazine interacts with a novel site in a GABA‐gated bacterial homologue.

**What does this study adds**

- The effects of clozapine on α5β3γ2 GABA receptors are consistent with orthosteric antagonism.
- Chlorpromazine does not displace [^3^H]muscimol and interacts with a novel site in α5 subunits.

**What is the clinical significance**

- Two or more distinctive mechanisms induce GABA current reduction by the tricyclic compounds.
- Inhibition of α5 subunit‐dependent current might contribute to clinically observed drug effects.

## 1 INTRODUCTION

Hippocampal dysfunction has long been considered to contribute to the pathophysiology of schizophrenia (Lieberman et al., 2018; Lodge & Grace, 2011; Nakahara et al., 2018). Post‐mortem studies in the brains of patients with schizophrenia suggest that hippocampal and prefrontal expression of GABA_A_ receptors is altered in a subtype‐selective manner (Skilbeck et al., 2007). The α5 GABA_A_ receptor subunit, which is characterized by its relatively limited distribution and high abundance in the hippocampus, has thus been in the focus of clinical and preclinical schizophrenia research (Marques et al., 2020; Xu & Wong, 2018). A recent PET study using [^11^C]Ro15‐4513, a radiotracer with high affinity to α5‐containing GABA_A_ receptor subtypes, found evidence for aberrant receptor levels in the hippocampus of patients with schizophrenia (Marques et al., 2020). Moreover, the study demonstrated a direct relationship between the expression of schizophrenia symptoms and hippocampal binding of [^11^C]Ro15‐4513. The quest for α5‐containing subtype‐preferring ligands has provided a number of compounds widely used in research (Etherington et al., 2017; Gill & Grace, 2014; Knust et al., 2009). These molecules exert allosteric modulatory effects that can range from GABA‐induced current enhancement or reduction to silent but competitive binding (Sigel & Ernst, 2018). Based on genetic and pharmacological studies, drugs which target α5‐containing GABA_A_ receptors have been under investigation as cognitive enhancers (Xu & Wong, 2018). Negative modulation of α5‐containing GABA_A_ receptors has been shown to promote hippocampal gamma oscillations, long‐term potentiation, and learning, as well as have antidepressant effects associated with restored synaptic strength in the form of increased glutamatergic excitatory activity (Atack et al., 2006; Glykys et al., 2008; Xu & Wong, 2018). Among the most recent developments was a clinical trial examining basmisanil, a compound exerting negative modulatory effects at α5‐containing GABA_A_ receptors, as an add‐on treatment for antipsychotic therapy aiming to alleviate cognitive impairment of patients with schizophrenia (https://clinicaltrials.gov/ct2/show/NCT02953639).

Not only GABA_A_ receptor targeting drugs such as benzodiazepines or sedative general anaesthetics elicit effects at these receptors by allosteric interaction sites, but a wide range of small molecules have been identified as GABA_A_ receptor modulators, including multiple antipsychotic and antidepressant medications not intentionally targeting these receptors (Squires & Saederup, 1988, 1998). One of those is clozapine (CLZ), a tetracyclic compound displaying relatively weak dopamine receptor antagonism. However, it shows outstanding antipsychotic efficacy and ameliorates negative and cognitive symptoms of schizophrenia without inducing unwanted extrapyramidal side effects (Attard & Taylor, 2012; Seeman, 2006). On the other hand, the effects of many antipsychotics, like chlorpromazine (CPZ), were mainly attributed to blockade of dopamine receptors and it has received only minor attention in terms of its effects on GABA_A_ receptors (Mozrzymas et al., 1999; Schwartz & Mindlin, 1988; Seeman, 1980; Yokota et al., 2002). In the 80s and 90s, the interactions of several antipsychotics with GABA_A_ receptors have been considered serious candidates for eliciting part of the therapeutic effects but were never studied in α5‐containing receptors (Squires & Saederup, 1988, 1993, 1997, 1998, 2000).

There is broad consensus that clozapine can reduce GABA elicited effects by direct interactions with GABA_A_ receptors. The mechanism remains unclear and the binding sites were never identified (Korpi et al., 1995; Michel & Trudeau, 2000; Squires & Saederup, 1997, 1999). In this work, we bridge this historical gap and examine the functional effects of clozapine and six chemically similar compounds in recombinantly expressed GABA_A_ receptors, including α5‐containing receptors. We demonstrate functional inhibition of GABA elicited currents. To further elucidate the molecular substrate of the observed effects, we investigate a novel intrasubunit binding site in the extracellular domain (ECD) of the α5 subunit, which has been described as a chlorpromazine site in the homologous GABA‐gated *Erwinia* ligand‐gated ion channel (ELIC) (Nys et al., 2016). Accordingly, we find to inhibit α5‐containing GABA_A_ receptors allosterically, but clozapine to be an orthosteric antagonist of this subtype.

## 2 METHODS

### 2.1 Functional testing with two‐electrode voltage clamp (TEVC) in *X. laevis* oocytes

Stock solution and buffers were prepared as described by Simeone et al. (2017). For the electrophysiological experiments, GABA was dissolved in NDE buffer [96 mM NaCl, 5 mM HEPES‐NaOH (pH 7.5), 2 mM KCl, 1 mM MgCl_2_, 1.8 mM CaCl_2_] with a concentration in order to achieve the appropriate EC concentration relevant to each experiment. In brief, all other compounds were dissolved in DMSO with a stock concentration of 100 mM (except clotiapine which was dissolved in a 25 mM stock concentration) and for further dilutions, the compounds were diluted in NDE plus GABA (EC_X_).

The mutated rat α5 GABA_A_ receptor subunit cDNA constructs were purchased from Eurofins Genomics (Ebersberg, Germany). The company performed the cloning by the use of site directed mutagenesis on a rat α5 insert in a pCI vector (RRID:Addgene_74230) which was provided by us. The following constructs were created: α5F53W, α5S189W, α5L196W, α5L222W and α5F53W;L222W (numbering without signal peptide) and were validated by double stranded DNA sequencing. One of those (Leu196, with the equivalent Ile in ELIC) was also mutated by Nys et al. (2016) and was found to cause a significant reduction in the response of GABA and no significant change in EC_50_.

In order to generate mRNA, all constructs were linearized, transcribed and purified as described previously (Simeone et al., 2017). For the microinjection, the RNA of αβ receptor combinations was mixed at 1:1 ratio and α1,2βγ receptors were mixed at 1:1:5 ratio, whereas α5βγ receptors were mixed at 3:1:5 ratio (γ2S variant). The approach used for subunit concatenation of α1β3γ2 GABA_A_ receptors has been described previously (Simeone et al., 2019). The dual (γ2β3) and triple (α1β3α1) constructs were injected at a ratio of 1:1 (Simeone et al., 2019). β2γ2 receptors were mixed with a 1:3 ratio, as described in Wongsamitkul et al. (2017). The RNA for the α5(mut)β3γ2 receptors was mixed at 3:1:5 ratio, as for the wild‐type α5β3γ2, with a final concentration of 70 ng μl^‐1^.

Healthy defolliculated oocytes (Ecocyte Biosciences, Dortmund, Germany) were injected with an aqueous solution of mRNA with a Nanoject II (Drummond, Broomall, PA, USA). The injected oocytes were incubated at 18°C (ND96 + antibiotic) for 2–3 days for αβ receptors and for 3–4 days for αβγ receptors before recording. Electrophysiological recordings were performed as specified in Simeone et al. (2017). A GABA concentration amounting to 5%–10% of maximum GABA currents is termed EC_5–10_ and 20%–30% of maximum GABA currents is EC_20–30_, 15%–30% of maximum GABA currents is EC_15–30_ etc.). All GABA concentrations used in the various experiments of this study are summarized in Table S10. In the major receptor isoform (Olsen & Sieghart, 2008) we successfully reproduced inhibitory effects on α1β2γ2 receptors at 100 μM clozapine (Asproni et al., 2002). Experiments with the neurosteroid tetrahydrodeoxycorticosterone (THDOC) were performed, with pre‐application of clozapine immediately before clozapine and THDOC co‐application. To ensure the incorporation of the γ2 subunit, diazepam was applied at the end of each measurement (~200% at 1 μM). For β2γ2 receptors, sufficient positive modulation by 50 μM etomidate was used a control (Wongsamitkul et al., 2017). All recordings were performed at room temperature at a holding potential of −60 mV using a Dagan TEV‐200A two‐electrode voltage clamp (Dagan Corporation) and a Turbo Tec‐03X npi amplifier.

### 2.2 Preparation of rat hippocampal membranes

In these experiments we used Sprague‐Dawley rats (Strain OFA, Oncins France Strain A), bred and maintained in the Institute of Biomedical Research, Medical University of Vienna (Himberg, Austria). Fifty‐one female rats (3–4 weeks old) were killed by decapitation, the 102 hippocampi removed quickly, flash frozen in liquid nitrogen and stored at −80°C until needed. Ethical review and approval was not required because the EU directive 210/63/EU, which is also reflected by the Austrian federal law Tierversuchsgesetz 2012, states that killing of animals solely for the use of their organs and tissues is not considered a ‘procedure’ and does not require specific approval. In six independent preparations, 15–18 hippocampi were homogenized with an Ultra‐Turrax rotor‐stator homogenizer for 30 s in ice‐cold homogenization buffer (10 mM HEPES, 1 mM EDTA, 300 mM sucrose) and centrifuged at 45,000 g at 4°C for 30 min. The pellet was resuspended in wash buffer (10 mM HEPES, 1 mM EDTA), incubated on ice for 30 min and centrifuged at 45,000 g at 4°C for 30 min. The pellet was stored at −80°C overnight and the next day washed three times by suspension in 50 mM Tris‐citrate buffer, pH = 7.1 and subsequent centrifugation, as described above. Membrane pellets were stored at −80°C until final use.

### 2.3 Radioligand membrane displacement assays

Frozen membranes were thawed, resuspended and incubated for 60 min at 4°C in a total of 500 μl of TC50/NaCl (50 mM Tris‐citrate pH = 7.1; 150 mM NaCl), various concentrations of the drug to be studied and 10 nM [^3^H]muscimol in the absence or presence of 10 mM GABA (to determine non‐specific binding; final DMSO‐concentration 1%). Membranes were filtered through Whatman GF/B filters and the filters were rinsed twice with 4 ml of ice‐cold 50 mM Tris/citrate buffer. Filters were transferred to scintillation vials and subjected to scintillation counting after the addition of 3 ml Rotiszint Eco plus liquid scintillation cocktail. The scintillation counter used is TriCarb 4910TR from Perkin Elmer.

The individual data points were performed in duplicate and repeated in three independent experiments. For the comparison of the degree of ligand displacement at 1 mM, five independent measurements were performed, each in duplicate.

### 2.4 Ligand similarity analysis, pharmacophore modelling and screening

For every ligand a conformer ensemble was generated using OMEGA 3.1.1.2 (OpenEye, RRID:SCR_014880; OpenEye Scientific Software, Santa Fe, NM, USA. http://www.eyesopen.com) (Hawkins et al., 2010) applying default settings for all parameters and output in SD‐format. Shape and colour similarity scores were calculated using ROCS 3.3.1.2 (OpenEye, RRID:SCR_014880) (Hawkins et al., 2007) with the ‐mcquery parameter set to true and applying default settings for all other parameters. The same combined multi‐conf. SD‐file of all ligands was specified both as input file for the query structures and the screened molecule database. The pairwise Shape Tanimoto, Colour Tanimoto and Tanimoto Combo scores calculated for a particular ligand were then extracted from the corresponding ROCS CSV output file that was generated for this ligand. Hierarchical clustering was performed by means of a small python script (Python Programming Language, RRID:SCR_008394; https://www.python.org/) using the clustering functionality provided by SciPy (Virtanen et al., 2020). For plotting the dendrogram the Matplotlib (MatPlotLib, RRID:SCR_008694; https://ieeexplore.ieee.org/document/4160265) package was used (Hunter, 2007). 2D scatter plots were generated in python using the multidimensional scaling (MDS) functionality provided by Scikit‐learn (https://scikit-learn.org/stable/; Pedregosa et al., 2012). The points, each representing one of the seven compounds, were coloured according to cluster membership and visualized by means of Matplotlib.

Ligand‐based pharmacophore models of the identified ligand clusters were generated using LigandScout 4.4 (LigandScout, RRID:SCR_014889; Inte:Ligand GmbH, Vienna, Austria; http://www.inteligand.com/ligandscout) (Wolber et al., 2006; Wolber & Langer, 2005). In the ligand‐based modelling perspective, all ligands constituting a cluster were added to the training‐set and then conformers were generated using iCon (LigandScout function; Poli et al., 2018) in FAST mode but with the RMSD threshold set to 0.35 to obtain denser conformer ensembles. Ligand‐based model generation was performed with the output pharmacophore type set to ‘Shared feature pharmacophore’ and default settings for all other parameters.

Structure‐based 3D pharmacophore modelling and subsequent screening of clozapine, imipramine, clotiapine, nortriptyline, levomepromazine and loxapine was performed using LigandScout 4.4. The compound pharmacophore screening database was generated using the idbgen module in LigandScout employing the ‘Best’ mode for conformer ensemble generation. Structure‐based pharmacophores were generated from the complexes 6HUG (picrotoxin site), 6X3S (bicuculline site) and 5LG3 (chlorpromazine‐bound ELIC) using LigandScout default settings in the structure‐based perspective. In the screening perspective, two screening runs with different stringency levels were carried out for each pharmacophore: (a) all query features have to be matched and (b) one arbitrary query feature may be omitted for hit identification. In both screening runs exclusion volume checks were enabled and the default scoring function ‘Pharmacophore‐Fit’ was used.

### 2.5 Computational modelling and docking

Alignments were generated with MOE (http://www.chemcomp.com) and Promals3D (http://prodata.swmed.edu/promals3d ). Files from the PDB (5LG3, 6A96) (Liu et al., 2018; Nys et al., 2016) were analysed as follows: Structural superpositions were performed with the PDBeFold webserver (Secondary Structure Matching, RRID:SCR_008365; http://www.ebi.ac.uk/msd-srv/ssm/) and further processed with MOE. Pocket volumes were calculated with Conolly, as implemented in MOE.

Molecular Docking was performed using GOLD 5.7.167 (chlorpromazine), and GOLD 2020.2.0 (clozapine) (GOLD, RRID:SCR_000188; https://www.ccdc.cam.ac.uk/solutions/csd-discovery/components/gold/) after appropriate preparation of protein and ligands. The ligands Ring‐NR1R2 was set flexible for all docking runs. MarvinSketch 19.9 (https://chemaxon.com/) with the protonation pKa function was used to prepare the ligand species for physiological pH (one for chlorpromazine, two for clozapine, see Figure S12).

6A96 was used as the wild type structure, and the mutants were introduced individually without further modifications using the MOE Protein Builder function. Chlorpromazine was docked into the site deduced from 5LG3: The centroid of the binding site for chlorpromazine was chosen by the position of the sulfur from the chlorpromazine of 5LG3 after it was superposed with the α5 subunit of 6A96. The binding site radius was set to 10 Å for both binding sites.

For the clozapine docking into the orthosteric site, the centroid of the binding site was chosen by the position of the nitrogen of the bicuculline of 6X3S after it was superposed with the β3 and α5 interface of 6A96.

On the protein, for the chlorpromazine docking, soft potentials have been set on the residues G187‐H195 (segment F) in 6A96 and the sidechains (α5V50, α5F53W, α5V56, α5V184, α5S189, α5L191, α5Y194, α5L196, α5F220 and α5L222W) were set flexible. For the clozapine docking, soft potentials have been set on the residues β3V199‐A204 (loop C) in 6A96 and the side chains α5D47, α5Y49, α5F68, α5R70, α5L121, α5L131, β3T133, β3Y157, β3F200 and β3Y205 were set flexible.

For chlorpromazine, two docking runs were performed, one with maximum diversity posing enforced for which 100 poses have been generated and one with default posing for which 300 poses were retained. For each protonation state of clozapine, 300 poses were generated with maximum diversity posing enforced. In each run, Goldscore (chlorpromazine) or CHEMPLP (clozapine) was used as the primary scoring function (default), and Chemscore (all from GOLD) for re‐scoring. The posing space from the diversity enforced runs was analysed based on the top 10 solutions of either scoring function, and related poses were clustered and pooled from both runs for chlorpromazine. Consensus score filtering led to three clusters of chlorpromazine poses in the top three positions. Representative poses of these clusters were subjected to energy minimization with MOE and depicted to visually analyse the effects of the mutants. For clozapine, a single run per protonation state with 300 poses each led to a sufficiently converged posing space, and the binding mode poses which share features with the bicuculline‐bound state were energy minimized with MOE with the Amber10:EHT forcefield. The best ranked pose was visualized (see Figure S12 for consensus scoring summary).

### 2.6 Data analysis and figure generation

The data and statistical analysis comply with the recommendations of the British Journal of Pharmacology on experimental design and analysis in pharmacology (Curtis et al., 2018). TEVC data was recorded and digitized using an Axon Digidata 1550 (and Axon Digidata 1550A) low‐noise data acquisition system (Axon Instruments, Molecular Devices, Wokingham, UK). Data acquisition was performed using pCLAMP v.10.5 (pClamp, RRID:SCR_011323; Molecular Devices). The same programme was used for the processing of representative traces, which were later imported to GraphPad Prism (v.6.) (GraphPad Prism, RRID:SCR_002798; San Diego, CA, USA) and visualized. A fraction of traces was analysed in a blinded fashion. Data were analysed using GraphPad Prism (v.6.) and plotted as concentration‐response curves or column graphs, as defined in Simeone et al. (2017). Figures of concentration–response curves and column graphs were generated using GraphPad Prism (v.6.). These curves were normalized and fitted by non‐linear regression analysis to the Equation Y = bottom + (top‐bottom)/(1 + [IC50/X]˄nH), where IC50 is the concentration of the compound that decreases the amplitude of the GABA‐evoked current by 50%, and nH is the Hill coefficient. Concentration‐response curves that did not reach saturation or where fits were not possible, were fitted by non‐linear regression using constrained fits of bottom to 0 or of Hill slope to 1 for best description of the data. The fit and constraint chosen is described in the figure legends. Structural images were generated using MOE, while images with pharmacophore models using LigandScout 4.4.

### 2.7 Data and statistical analysis

The assumption of normality around reported mean values was confirmed using the Shapiro–Wilk test with an alpha value of 0.05. To determine the significance in variance of the results obtained from three or more groups, one‐way ANOVA with Geisser–Greenhouse correction was performed followed by a Dunnett's multiple comparisons test. When the data do not assume a normal distribution, the non‐parametric one‐way ANOVA (Kruskal–Wallis test) was used followed by a Dunn's multiple comparisons test. All data are expressed as mean ± SEM. Differences between two groups were analysed using a two‐tailed Student's t‐test. One sample t‐test was performed in order to determine statistical significance of each mean response from control current (100%). The false discovery rate (FDR) for these tests was controlled, and *P*‐values were adjusted using the Bejamini–Hochberg method with a discovery rate (Q value) of 0.05 (where #*P* < 0.05). A *P*‐value less than 0.05 was considered statistically significant and only one level of statistical significance was used throughout the study. All statistical tests that have been used, and applied to sample sizes in the study, are indicated in the figure legends. The n number stated represents the number of single oocyte experiments. The exact n values are reported by the individual values shown in all scatter plot bar graphs, as well as in the figure legends and tables in the supporting information. All data subjected to statistical analysis have a group size of (n) ≥ 5. Statistical analysis was performed using GraphPad Prism (v.6.).

### 2.8 Materials

*Xenopus laevis* oocytes were commercially purchased from Ecocyte Biosciences (Dortmund, Germany). Compounds purchased from Sigma Aldrich (Vienna, Austria) were GABA (A2129‐100g), chlorpromazine (C8138‐5g) and imipramine (I7379‐5g). Loxapine (L106‐100mg) was from Biomedica Medizinprodukte (Vienna, Austria) and clozapine (RD 0444/50) was from THP Medical Products (Vienna, Austria). Levomepromazine (MCE‐HY‐B1693‐100mg), nortriptyline (T1327‐200mg) and clotiapine (SACSC‐200404A) were from Szabo‐Scandic Handels (Vienna, Austria). [^3^H]muscimol (NET574250UC) was purchased from PerkinElmer ( Beaconsfield, United Kingdom). All other chemicals were purchased from Sigma Aldrich.

### 2.9 Nomenclature of targets and ligands

Key protein targets and ligands in this article are hyperlinked to corresponding entries in http://www.guidetopharmacology.org and are permanently archived in the Concise Guide to PHARMACOLOGY 2021/22 (Alexander, Christopoulos et al., 2021; Alexander, Mathie et al., 2021).

## 3 RESULTS

### 3.1 Functional profiles of clozapine and chlorpromazine on different GABA_A_ receptor subtypes

**FIGURE 1:**
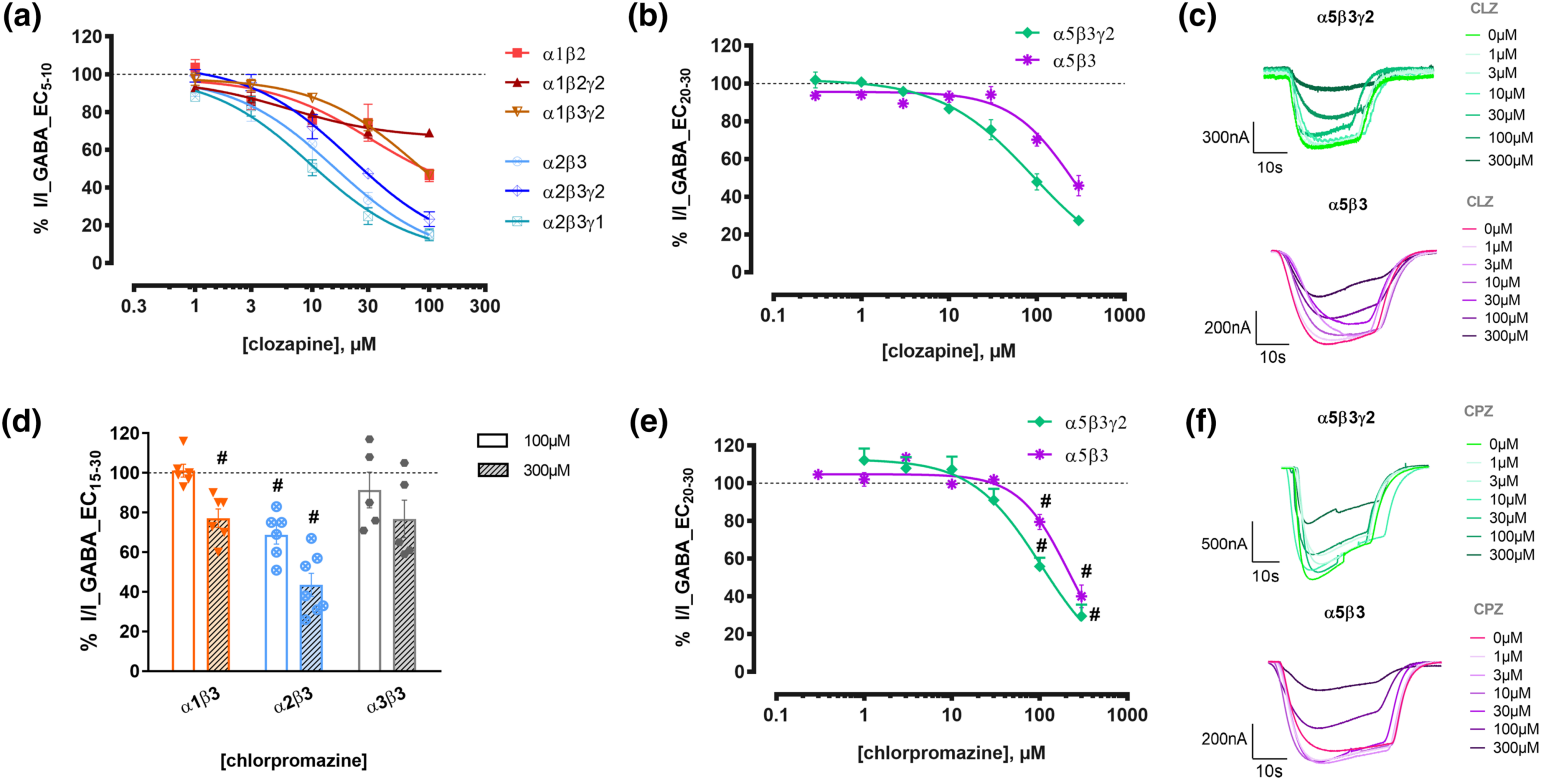
Functional inhibition by clozapine and chlorpromazine of different GABA_A_ receptor subtypes. (a) Clozapine concentration‐response effects elicited by an EC_5–10_ GABA concentration in α1β2 (n = 5), α1β2γ2 (n = 5–7), α1β3γ2 (n = 6–9), α2β3 (n = 5–11), α2β3γ2 (n = 6) and α2β3γ1 (n = 5) receptors. The effects we observed by co‐application of 100 μM clozapine with GABA EC_5–10_ are summarized in Figure S1 (including effects on the additional subtype α1β3). Data were fitted to the Hill equation using non‐linear regression (fixed slope of 1) and are shown as means ± SEM. Representative traces can be found in Figure S2. (b,e) Clozapine (b) and chlorpromazine (e) concentration‐response effects elicited by an EC_20–30_ GABA concentration in α5β3γ2 receptors (n = 5–11 and n = 7–13) and in α5β3 receptors (n = 5–6 and n = 6). Data were fitted to the Hill equation using non‐linear regression (fixed bottom of 0), and are shown as means ± SEM. (c,f) Representative traces from electrophysiological recordings of clozapine (c) and chlorpromazine (f) co‐applied with GABA in α5β3γ2 and α5β3 receptors, corresponding to panels (b) and (e). The dotted line is used to visualize the baseline (100%) of control current. The IC_50_, logIC_50_, Hill slope and maximum efficacy values corresponding to panels (a), (b) and (e) are in Tables S1, S2 and S3. (d) Modulation of currents elicited by an EC_15–30_ GABA concentration by 100 and 300 μM chlorpromazine in α1β3 (n = 6 and n = 6), α2β3 (n = 6 and n = 7) and α3β3 (n = 5 and n = 5) receptors. Data shown are individual values with means ± SEM; control currents are shown as the dotted line across the graph. #*P* < 0.05, significantly different from control current; one sample t‐test with corrected for multiple comparisons using the false discovery rate method of Benjamini and Hochberg, with a discovery rate of 0.05. The response with 100 μM in α2β3, α5β3 and α5β3γ2 receptors was significantly different from control, while with 300 μM the responses in α1β3, α2β3, α5β3 and α5β3γ2 receptors were significantly different from control

First, we examined the effects of clozapine and chlorpromazine on recombinantly expressed GABA_A_ receptors. We performed functional testing of the drugs' effects in a panel of subunit combinations with emphasis on subtypes reported as candidate targets for alleviating some schizophrenia symptoms, namely, α2 and α5 subunit‐containing GABA_A_ receptors (Xu & Wong, 2018). In earlier experiments where a different subtype panel was investigated, inhibitory as well as biphasic modulation of radioligand binding was observed (Korpi et al., 1995), prompting us to use a low GABA concentration (EC_5–10_) for the initial functional assessment. Only current reduction was seen in the tested range with clozapine (1‐100 μM) and no enhancement or biphasic effects were observed (Figure 1a). Inhibition in the tested α1‐containing assemblies was less pronounced, than those in α2‐ and α5‐containing assemblies (Figures 1a and S1a,b). Additionally, the α5β3 dose response curve was right‐shifted compared to α5β3γ2 (Figure 1b,c). The current reduction approaches plateau at around 100 μM for five subunit combinations, namely, α1β2γ2, α2β3, α2β3γ2, α2β3γ1 and α5β3γ2, but the extent of inhibition varied from 69% to 15% (Figures 1a and S1a). Chlorpromazine displays actions similar to those of clozapine in α5β3γ2 and α5β3, and screening at 100 μM in α1β3 and α2β3 revealed weaker current inhibition compared with clozapine (Figure 1d,e,f). Both compounds fail to inhibit currents in the α3β3 subunit combination (Figures 1d and S1b).

Neurosteroids, such as THDOC, are known to directly activate GABA_A_ receptors (Wohlfarth et al., 2002). We also examined whether clozapine could inhibit neurosteroid‐activated currents, similarly to known orthosteric antagonists (Puia et al., 1990). In our experiments, clozapine did not inhibit THDOC‐gated currents in α1β3 GABA_A_ receptors (Figure S1c). Moreover, in an effort to assess the effect of the α‐subunit on the observed effects, we compared clozapine responses between α5β2γ2 and β2γ2 receptors. The latter receptors have been previously described and were found to be GABA‐gated, as well as being modulated by diazepam and etomidate (Wongsamitkul et al., 2017). Removal of the α5 subunit from the receptor assembly eliminated a significant part of the effect (Figure S1d) and chlorpromazine was completely inactive in β2γ2 receptors (Figure S1e). Similar to the diversity of effects observed in a [^35^S]TBPS modulation study (Korpi et al., 1995), each subunit isoform influences the net effect of clozapine on a given subunit combination.

### 3.2 Investigation of additional tricyclic compounds

**FIGURE 2:**
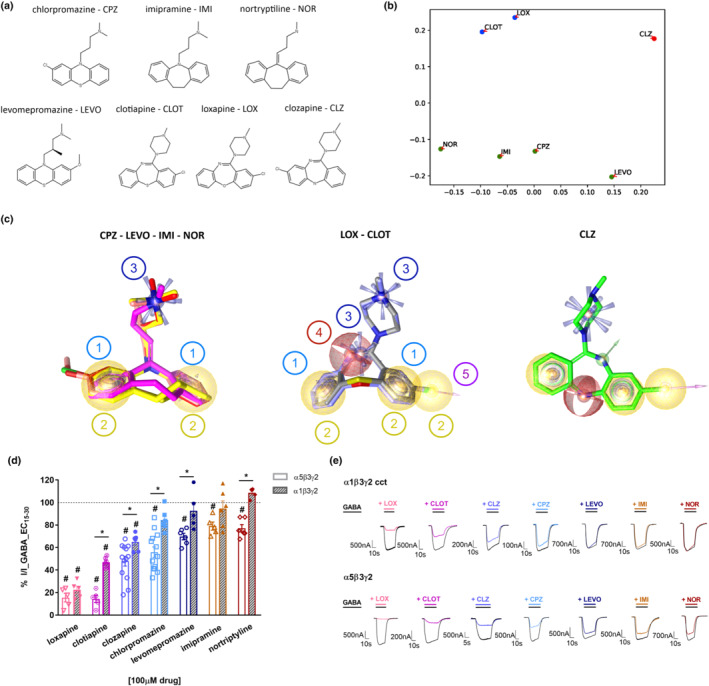
Chemical structures and selected properties of all drugs investigated in this study. (a) Chemical structures of clozapine (CLZ), chlorpromazine (CPZ), levomepromazine (LEVO), imipramine (IMI), nortriptyline (NOR), loxapine (LOX) and clotiapine (CLOT). (b) 2D scatter plot of the compounds where the proximity of the points correlates with the corresponding Tanimoto combo similarity scores calculated by ROCS and the axes reflect a dimensionless distance. Figures S3 and S4 show the raw data and the results of hierarchical clustering. Individual shape, colour and combo scores are shown in Table S4. (c) Ligand‐based shared feature pharmacophores generated by LigandScout of the ligand groups that emerged from panel (b). Features: 1 – aromatic (blue donuts), 2 – hydrophobic (yellow spheres), 3 – positive ionizable (blue stars/rays), 4 – hydrogen bond acceptor (red sphere), 5 – halogen bond donor (magenta arrow). (d) Modulation of currents elicited by an EC_15–30_ GABA concentration by 100 μM CLZ (n = 13), CPZ (n = 13), NOR (n = 6), IMI (n = 5), LEVO (n = 6), LOX (n = 5) and CLOT (n = 5) in α5β3γ2 and in concatenated α1β3γ2 wild‐type receptors (n = 6, n = 6, n = 5, n = 6, n = 5, n = 5, n = 6, respectively). Subset of data in α5β3γ2 receptors for CLZ from Figure 1d, reproduced here for the comparison with α1β3γ2 receptors. Data for each receptor subtype are shown as individual values with means ± SEM; control currents are shown as the dotted line across the graph. The mean response in α1β3γ2 receptors was not significantly different from control current for chlorpromazine, levomepromazine, imipramine and nortriptyline. The mean response in α5β3γ2 receptors was significantly different from control current for all drugs. #*P* < 0.05, significantly different from control current; one sample t‐test. *P < 0.05, significant differences between α5β3γ2 and α1β3γ2 receptors; two‐tailed Student's t‐test; both t‐tests were corrected for multiple comparisons using the false discovery rate method of Benjamini and Hochberg (discovery rate of 0.05). (e) Representative traces from electrophysiological recordings of LOX, CLOT, CLZ, CPZ, LEVO, IMI and nortriptyline co‐applied with GABA in α1β3γ2 (concatenated) and α5β3γ2 receptors

Different studies accumulated over several years showed that clozapine and several other antipsychotic and antidepressant drugs are full or partial inhibitors of GABA_A_ receptors (Squires & Saederup, 1987, 1988, 1997, 1998, 2000). Most of the earlier work was done in membrane preparations from rodent brains. We, therefore, chose to test a selection of compounds that were already investigated by Squires and Saederup in the 80s and 90s but in defined, recombinantly expressed subunit combinations. Additional tricyclic compounds with chemical structures comparable to clozapine were tested, namely, levomepromazine, imipramine, nortriptyline, loxapine and clotiapine (Figure 2a).

All of these compounds share a cyclic scaffold composed of two benzene rings flanking a central, non‐aromatic 6‐ or 7‐membered ring with a substituent that carries a terminal amino group. For a more in‐depth investigation of structural and stereoelectronic similarities between the selected compounds, we performed pairwise shape alignments using the software ROCS (Hawkins et al., 2007). Three types of scores were computed and analysed further, namely, pure shape similarity (shape), overlap of shared features (colour), and a combination score (combo) which considers both shape and feature overlap. 2D scatter plots of the compounds via a multidimensional scaling procedure of the similarity scores visualize the calculated scores of each compound pair (Figures 2b and S4). The visual analysis of the scatter plots revealed two groups, namely, chlorpromazine, imipramine, nortriptyline, levomepromazine and loxapine, clotiapine, clozapine (Figure 2b). For a more in‐depth investigation of ligand similarities in terms of common chemical features and the resulting receptor interaction capabilities, we generated ligand‐based pharmacophore models for both ligand groups using the software LigandScout (Wolber et al., 2006; Wolber & Langer, 2005). The group comprising chlorpromazine‐ levomepromazine‐ imipramine‐nortriptyline has two hydrophobic, two aromatic and one positive ionizable feature (Figure 2c). Loxapine and clotiapine contain several additional features, while clozapine shares with loxapine and clotiapine, three hydrophobic, two aromatic, one positive ionizable and one halogen bonding feature, where not all can be aligned simultaneously (Figures 2c and Figure S5). All drugs have two hydrophobic, one aromatic and one positive ionizable feature in common. The overall shape similarity is high across all seven compounds, and thus suggestive of shared targets while differences in features may reflect some non‐overlapping targets.

While all these compounds (except levomepromazine) were known to interact with GABA_A_ receptors, their functional effects have never been compared systematically. We thus examined their effects on GABA currents in α1β3γ2 (concatenated; Simeone et al., 2019) and α5β3γ2 receptors (Figure 2d,e). All of them diminished GABA‐elicited currents in α5β3γ2 receptors, and all but loxapine and imipramine elicited greater peak current inhibition in the α5‐containing subtype. The chlorpromazine‐ levomepromazine‐ imipramine‐ nortriptyline group had no significant effects on the α1β3γ2 receptors at 100 μM.

### 3.3 Computational exploration of candidate binding sites

**FIGURE 3:**
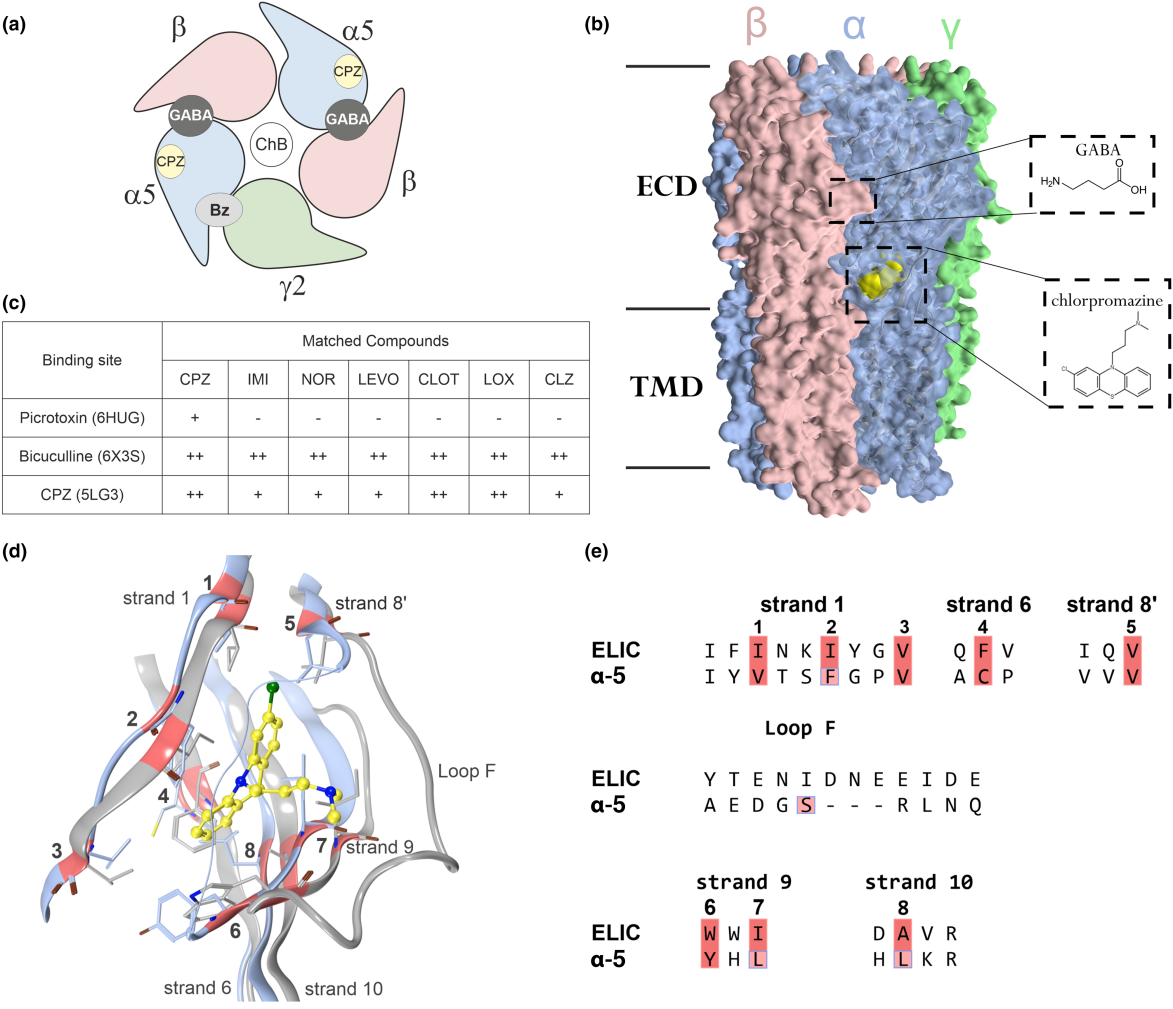
Candidate binding sites for the current reduction elicited by the tricyclic compounds and the putative chlorpromazine pocket in the α5 subunit. (a) Cartoon view of a receptor with the canonical subunit arrangement, the pointed side of the subunit is the principal side. The localization of the GABA/bicuculline sites (orthosteric sites) and the high affinity Bz‐sites are at subunit interfaces. The channel blocker (ChB) site is localized in the pore domain. The candidate site for chlorpromazine (CPZ) in the α5 subunit is shown as a yellow circle, the α‐subunit in blue, β in red and γ in green. (b) Space filling representation of a heteropentameric GABA_A_ receptor (PDB ID: 6A96) with chlorpromazine docked into the candidate binding site in yellow space filling representation. The insets display GABA and CPZ structures and binding site localizations. Sequences with binding site forming amino acids and a comparison among alpha isoforms are provided in Figure S6. (c) Table of the pharmacophore screening results into the selected bound state structures: ++ = all features matched, + = match with one omitted feature, − = no match. (d) Homology between the CPZ site in ELIC (5LG3) and the corresponding pocket in the α5 subunit of 6A96. 3D superposition of an α5 (light blue) subunit of 6A96 and ELIC (5LG3 in grey), respectively. Strands 1, 6 and 10 are highly conserved, and the hydrophobic amino acids forming the large deep portion of the pocket overlap closely, while loop F is longer in ELIC. (e) Partial sequence alignment of the pocket forming protein segments of ELIC with the GABA_A_ receptor α5 subunit. The hydrophobic pocket core positions are highlighted red and correspond with the red ribbon markings in panel (d). The amino acids highlighted in pink boxes indicate sites chosen for mutational analysis (Figure 4a)

GABA‐elicited currents can be reduced by multiple different mechanisms, specifically by direct pore block at the picrotoxin site, by competitive antagonism at the orthosteric site akin to bicuculline, and by negative allosteric modulation from different allosteric sites such as the Bz‐site, for which a γ‐subunit is needed (Figure 3a). The observation that clozapine and chlorpromazine do not need the γ‐subunit for effective reduction of GABA currents rules out the Bz‐site for their action, in line with previous work (Korpi et al., 1995).

We turned to structural data from the Protein Data Bank (PDB) in order to perform a computational exploration of the remaining candidate binding sites. GABA_A_ receptor structures in picrotoxin‐ and bicuculline‐bound states are available (Kim et al., 2020; Masiulis et al., 2019). In a search for homologous proteins in complex with any of our test ligands, a chlorpromazine‐bound structure of a homologous, bacterial GABA‐gated pentameric channel, namely, ELIC (Nys et al., 2016) was found. The chlorpromazine pocket observed in the bacterial superfamily member has been previously suggested to be compatible with homology models of GABA_A_ receptors (Puthenkalam et al., 2016), where it is located near the disulfide bridge in the packing core between the ECD inner and outer sheets (Figure 3b).

First, we performed a pharmacophore‐based screening of the investigated compounds using structure‐based pharmacophores generated for picrotoxin‐ and bicuculline‐bound states of GABA_A_ receptors and the chlorpromazine‐bound ELIC (Figure 3c). The screening runs were performed at two different levels of stringency: (a) all features have to be matched and (b) one feature may be omitted to obtain a match. No matches were found for the pharmacophore of the picrotoxin site at high stringency, and chlorpromazine matched with one omitted feature. For the bicuculline/GABA site, all ligands match in the stringent screening run. For the chlorpromazine site in ELIC, loxapine, chlorpromazine and clotiapine match in the stringent run, while the remaining ligands match with less stringent settings. Due to these results, we moved on to further explore the chlorpromazine site and the orthosteric site. As α‐subunits strongly influence the net effect elicited by chlorpromazine or clozapine, we chose to investigate the novel candidate chlorpromazine site in the α5 subunit, taking advantage of a recently published cryo‐EM structure of α5β3 (Figure 3d) (Liu et al., 2018).

The binding site occupied by chlorpromazine in ELIC (Nys et al., 2016) is formed by hydrophobic sidechains located on strands 1, 6 and 10 and capped by the back of segment (loop) F, which provides both hydrophobic and polar interactions. Chlorpromazine interacts with the pocket mainly via van der Waals contacts of the tricyclic core, while the sidechain forms polar interactions with hydrophilic groups of segment F (Nys et al., 2016). Superposition with the available structure of the α5 subunit indicates good overlap of the strands, and very little overlap for the segment F (Figure 3d,e).

### 3.4 Mutational analysis of the putative chlorpromazine binding site in the α5 subunit

**FIGURE 4:**
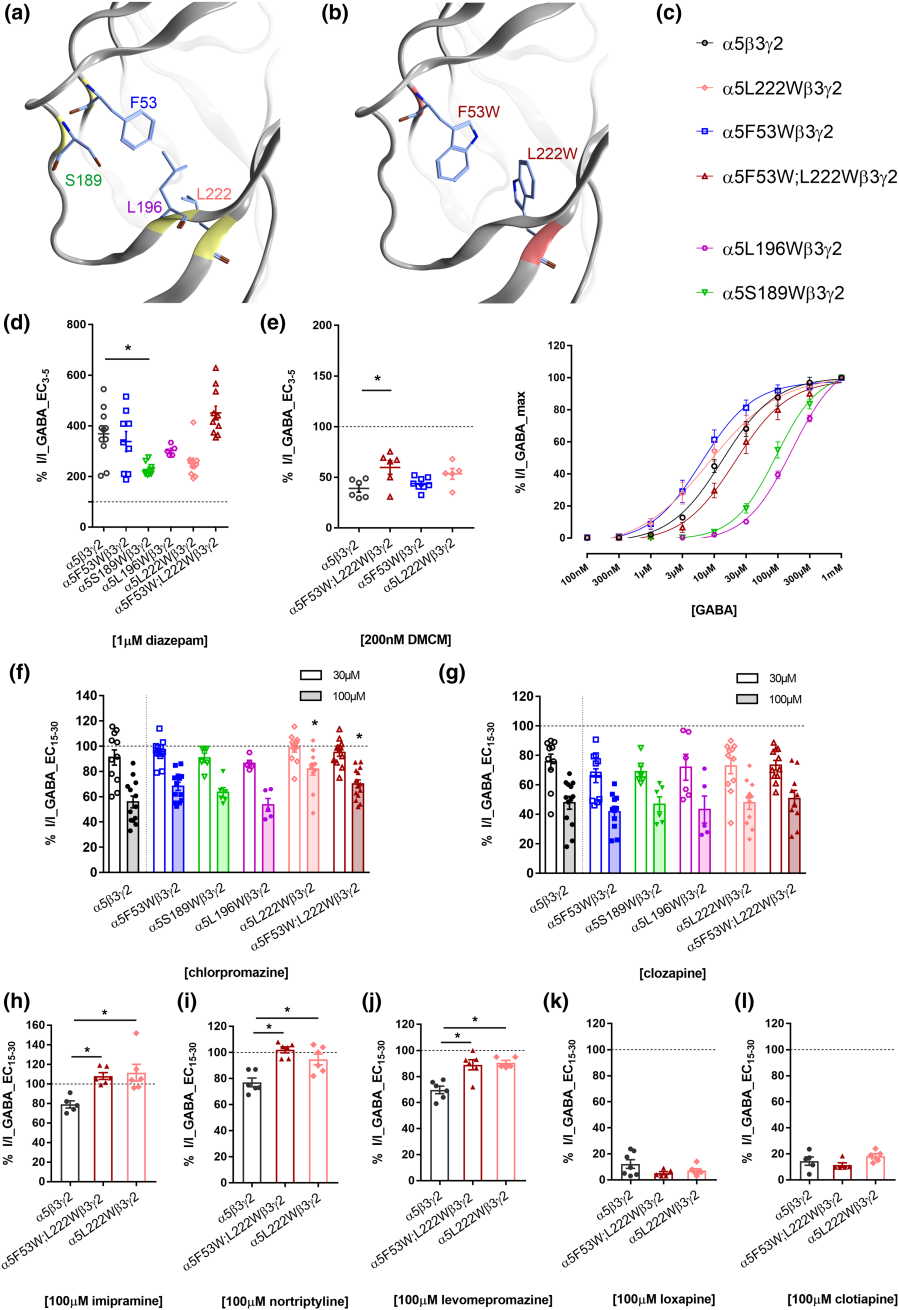
Mutational analysis of the putative binding site in the α5 subunit impacts on some of the drugs' effects. (a) Binding site region of the α5 subunit of the GABA_A_ receptor, highlighting the residues subjected to mutational analysis, namely, F53, S189, L196 and L222 where the colour codes of the labels match panel (c). (b) Structural rendering of F53W;L222W. The estimated volume of the binding site is reduced in the double mutant by up to 46%, depending on rotameric states. (c) GABA dose response curves in α5β3γ2 (n = 5), α5F53Wβ3γ2 (n = 6), α5S189Wβ3γ2 (n = 6), α5L196Wβ3γ2 (n = 6), α5L222Wβ3γ2 (n = 6) and α5F53W;L222Wβ3γ2 (n = 5) receptors. Data were normalized and fitted to the Hill equation using non‐linear regression and are shown as means ± SEM. The EC_50_, logEC_50_, Hill slope and amplitude at 1 mM values are listed in Table S5. (d, e) modulation of currents elicited by an EC_3–5_ GABA concentration by 1 μM diazepam (d) and 200 nM DMCM (e) in α5β3γ2 (n = 11 and n = 6), α5F53Wβ3γ2 (n = 9 and n = 8), α5S189Wβ3γ2 (n = 9), α5L196Wβ3γ2 (n = 5), α5L222Wβ3γ2 (n = 10 and n = 5) and α5F53W;L222Wβ3γ2 (n = 11 and n = 6) receptors. Sufficient positive allosteric modulation by 1 μM diazepam and negative allosteric modulation by 200 nM DMCM was achieved for all tested cells (≥200% and ≤50%, respectively). Data are shown as individual values with means ± SEM. For DMCM, *P < 0.05, significant differences between mutated and wild‐type receptors: one‐way ANOVA followed by Dunnett's multiple comparisons test. For diazepam, *P < 0.05, significant differences between mutated and wild‐type receptors; non‐parametric one‐way ANOVA (Kruskal–Wallis test) followed by Dunn's multiple comparisons test. (f, g) Modulation of currents elicited by an EC_15–30_ GABA concentration by 30 and 100 μM CPZ (f), as well as by 30 and 100 μM clozapine (CLZ; g) in α5β3γ2 wild‐type (n = 13 for 100 μM CPZ and n = 13 for 100 μM CLZ, n = 11 for 30 μM CPZ and n = 10 for 30 μM CLZ), α5F53Wβ3γ2 (n = 13 for 100 μM CPZ and n = 11 for 100 μM CLZ, n = 13 for 30 μM CPZ and n = 11 for 30 μM CLZ), α5S189Wβ3γ2 (n = 6 for 100 μM CPZ and n = 6 for 100 μM CLZ, n = 6 for 30 μM CPZ and n = 6 for 30 μM CLZ), α5L196Wβ3γ2 (n = 5 for 100 μM CPZ and n = 5 for 100 μM CLZ, n = 5 for 30 μM CPZ and n = 6 for 30 μM CLZ), α5L222Wβ3γ2 (n = 11 for 100 μM CPZ and n = 11 for 100 μM CLZ, n = 11 for 30 μM CPZ and n = 11 for 30 μM CLZ) and α5F53W;L222Wβ3γ2 (n = 14 for 100 μM CPZ and n = 10 for 100 μM CLZ, n = 10 for 30 μM CPZ and n = 11 for 30 μM CLZ) mutated receptors. Columns for each receptor subtype depict mean ± SEM. *P < 0.05, significant differences between mutated and wild‐type receptors; non‐parametric one‐way ANOVA (Kruskal–Wallis test) followed by Dunn's multiple comparisons test. (h–l) Modulation of currents elicited by an EC_15–30_ GABA concentration by 100 μM imipramine (IMI; n = 6) (h), nortriptyline (NOR; n = 6) (i), levomepromazine (LEVO; (n = 6) (j), loxapine (LOX; n = 5) (k) and clotiapine (CLOT; n = 5) (l) in α5F53W;L222Wβ3γ2 and by 100 μM IMI (n = 6) (h), NOR (n = 6) (i), LEVO (n = 5) (j), LOX (n = 6) (k) and CLOT (n = 5) (l) in α5L222Wβ3γ2 mutated receptors, compared to α5β3γ2 wild‐type receptors. All drug effects in wild‐type receptors as in Figure 2d are reproduced for direct comparison. Data are shown as individual values with means ± SEM. *P < 0.05, significantly different as indicated; one‐way ANOVA followed by Dunnett's multiple comparisons test

Encouraged by the good superposition of the chlorpromazine‐bound structure and the α5 subunit, four mutations in the α5 subunit were chosen based on pocket forming residues and their proximity to the ligand (Figure 4a). Tryptophan residues were introduced individually into the four sites and in one double mutant in order to diminish the pocket volume (Figure 4a,b).

In subsequent experiments, each α5 subunit mutant was co‐expressed individually with β3 and γ2, forming α5(mut)β3γ2 receptors. The GABA dose response curves of α5F53Wβ3γ2, α5L222Wβ3γ2 as well as α5F53W;L122Wβ3γ2 were matching the wild‐type, in comparison to the other two that were right‐shifted (Figure 4c). Diazepam effects (1 μM) were also examined in all mutated receptors and were above ~200% in wild‐type and mutated receptors, which ensures the incorporation of the γ2 subunit (Figure 4d). The known Bz‐site negative modulator DMCM was used as an additional control for non‐specific effects of the mutants in α5F53Wβ3γ2, α5L222Wβ3γ2 as well as α5F53W;L122Wβ3γ2. Only the double mutant displays a small but significant alteration in the DMCM modulation (Figure 4e). Effects of the individual mutations on current reduction by 100 μM chlorpromazine were significant for α5L222Wβ3γ2 and α5F53W;L122Wβ3γ2, and none induced significant changes for clozapine (Figure 4f,g). The most informative mutant was α5L222Wβ3γ2, with normal responses to GABA, diazepam and DMCM. Thus, the α5L222W mutant and the double mutant α5F53W;L122W were used to screen the remaining compounds for change in effect (Figure 4h–l). Loxapine, clotiapine and clozapine were not influenced by either mutant, while for chlorpromazine, levomepromazine, imipramine and nortriptyline, the inhibition was reduced in both mutants (Figure 4f,g and h–l). In order to ensure that we did not overlook differences for loxapine and clotiapine at compound concentrations that elicit a lower degree of inhibition, we repeated the experiments at additional compound concentrations and saw no effect of the double mutant (Figure S8).

**FIGURE 5:**
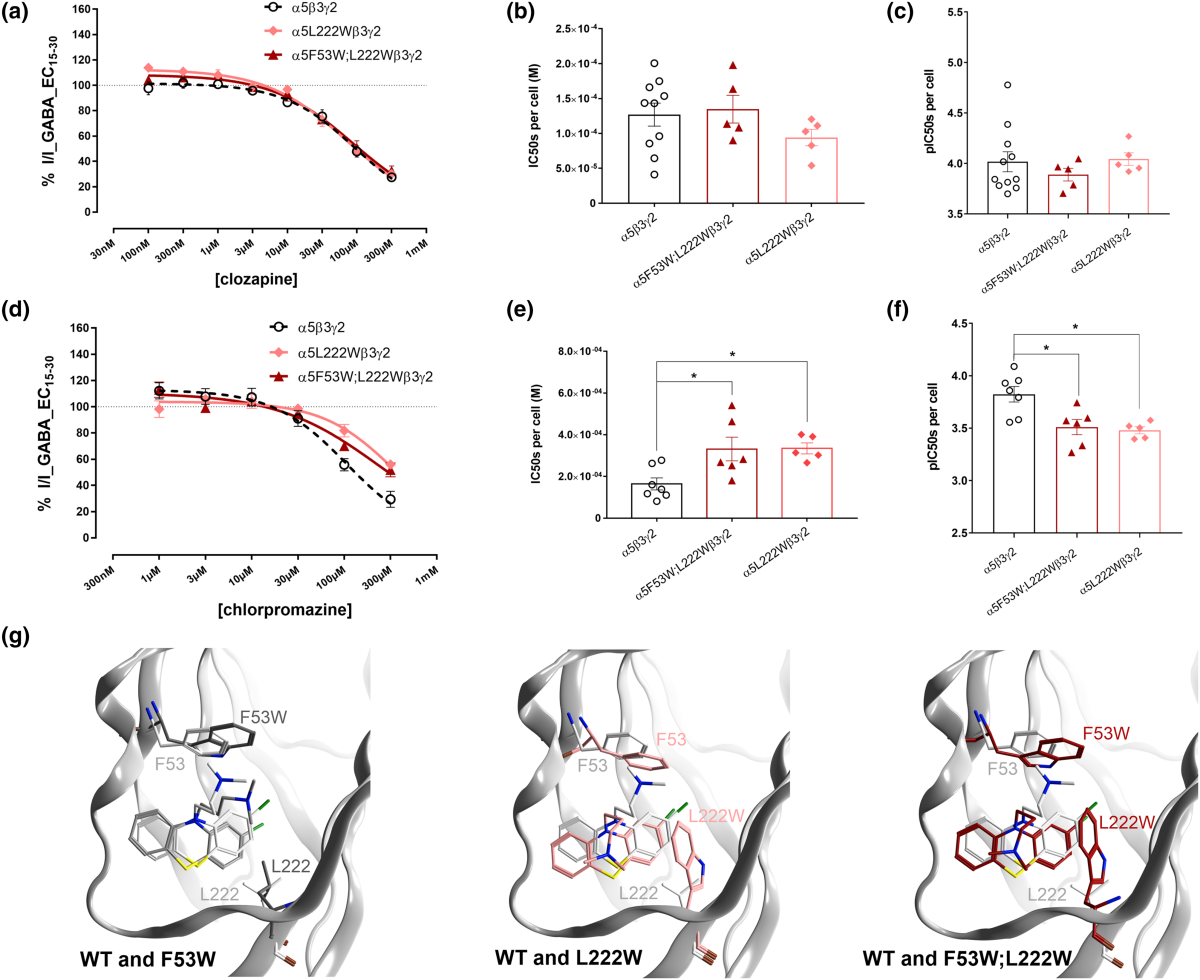
Mutational analysis of the effects of the putative binding site in the α5 subunit on the actions of chlorpromazine, but not those of clozapine (a, d). Concentration‐response curves for clozapine (a) and chlorpromazine (c) in α5β3γ2, α5L222Wβ3γ2 and α5F53W;L222Wβ3γ2 receptors (α5F53Wβ3γ2 receptors in Figure S9). Data were normalized and fitted to the Hill equation using non‐linear regression (fixed bottom of 0) and data are shown as mean ± SEM. The precise n numbers, as well as the IC_50_, logIC_50_, Hill slope and maximum efficacy values are in Tables S6 and S7. Concentration‐response curves in α5β3γ2 receptors are represented with dotted lines, as shown in Figure 1 and reproduced here for easier comparison. (b, e) The IC_50_ values for clozapine (b) and chlorpromazine (e) from concentration‐response assays in α5β3γ2, α5L222Wβ3γ2 and α5F53W;L222Wβ3γ2 receptors by fitting data of each cell individually. (c,f) The corresponding pIC_50_ values for clozapine (c) and chlorpromazine (f) dose response effects in α5β3γ2, α5L222Wβ3γ2 and α5F53W;L222Wβ3γ2 receptors by fitting data of each cell individually. Data are shown as individual values with means ± SEM. *P < 0.05, significantly different as indicated; one‐way ANOVA followed by Dunnett's multiple comparisons test. (g) Representative, energy minimized molecular docking poses of chlorpromazine in α5β3γ2 (white), α5F53Wβ3γ2 (grey), α5L222Wβ3γ2 (pink) and α5F53W;L222Wβ3γ2 (red) receptors

As the α5L222W mutant and the double mutant α5F53W;L122W altered the effects of chlorpromazine, but data for clozapine were inconclusive, we investigated our compounds of major interest— clozapine and chlorpromazine—over a concentration range (Figures 5a–f and S9). The α5F53W mutation did not influence the IC_50_ of either compound (Figure S9). None of the mutants altered the IC_50_ of clozapine (Figure 5a–c), while the IC_50_ of chlorpromazine (functional inhibition) was right‐shifted in α5L222Wβ3γ2 and in the double mutant α5F53W;L122Wβ3γ2 (Figure 5d–f).

The effect of the double mutant was expected to be stronger, based on pocket volume computation, prompting a more detailed follow up on a possible structural hypothesis for the small change in IC_50_. Docking of chlorpromazine into the four investigated pockets (wild‐type and the three mutants depicted in Figure 5g) resulted in several good candidate binding modes based on consensus scoring (Figure S10). Thus, docking suggests that chlorpromazine can be accommodated by the pocket in the wild type and the mutated pockets. The structural hypothesis which is most in line with no effect of the α5F53W mutant and an equal right shift for α5L222W and the double mutant α5F53W;L122W is displayed in Figure 5g. Other candidate binding modes including one that is similar to the 5LG3 structure are shown in Figure S10, along with their putative interaction features. In total, the data from the computational and mutational analysis suggests that a chlorpromazine site, homologous to the one described in ELIC, is present in the α5 subunit of GABA_A_ receptors.

### 3.5 Investigation of orthosteric site usage

**FIGURE 6:**
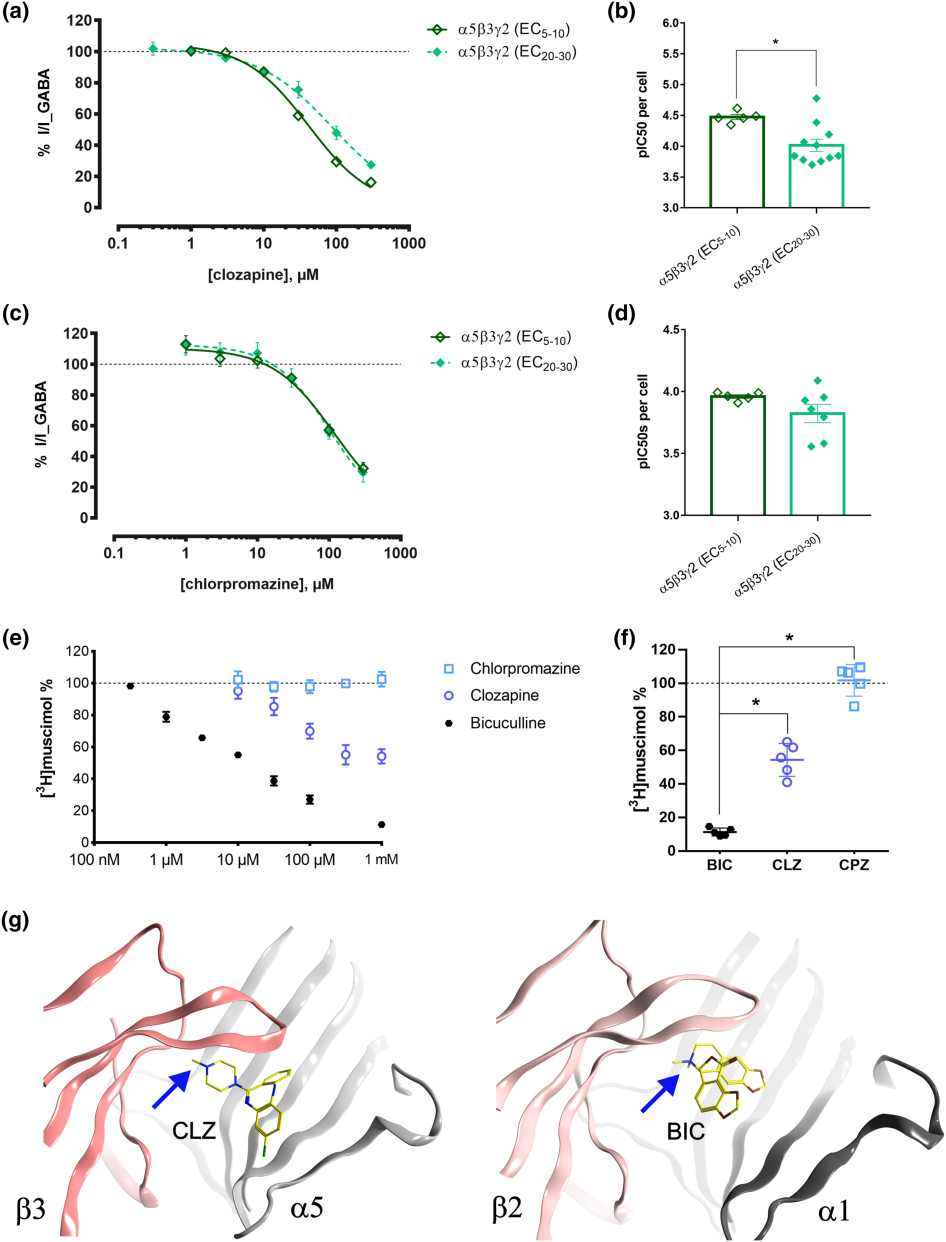
Exploration of orthosteric site usage by clozapine and chlorpromazine. Concentration‐response curves for clozapine (a) and chlorpromazine (c) in the presence of an EC_20–30_ and an EC_5–10_ GABA concentration in α5β3γ2 receptors. The dotted line is used to show the baseline (100%) of control current. Data were fitted to the Hill equation using non‐linear regression (fixed bottom of 0) and are shown as means ± SEM. (b,d) The pIC50 values for clozapine (b) and chlorpromazine (d) concentration‐response effects at EC_5–10_ (n = 5 and n = 5) and EC_20–30_ (n = 11 and n = 7) GABA concentration in α5β3γ2 receptors by fitting data of each cell individually. Results are shown as individual values with means ± SEM. *P < 0.05, significantly different as indicated; two‐tailed Student's t‐test. The IC_50_, logIC_50_, Hill slope and maximum efficacy values corresponding to panels (d) and (f) are in Tables S2 and S3. (e) Inhibition of binding of [^3^H]muscimol to rat hippocampal membrane GABA_A_ receptors (n = 3–5). Membranes were incubated with 10 nM [^3^H]muscimol in the presence of various concentrations of the displacing ligand. 100% is the amount of radioligand bound in the presence of 1% DMSO. Data shown are mean ± SEM of three independent experiments performed in duplicates each (for the concentrations <1 mM) and five independent experiments performed in duplicates each (for 1 mM). Visual inspection and sigmoid fitting indicated that the displacement points are not described by a single sigmoid function, as would be expected due to different affinities for the diversity of subtypes that are present in hippocampal membranes. Therefore, the individual points are displayed without fitting. (f) Inhibition of binding of [^3^H]muscimol to rat hippocampal membrane GABA_A_ receptors at 1 mM bicuculline (BIC), chlorpromazine (CPZ) and clozapine (CLZ). Hippocampal membranes from five independent membrane preparations were incubated with 10 nM [^3^H]muscimol in the presence of 1 mM of displacing ligand in five independent experiments performed in duplicates each. Result are shown as individual values with means ± SEM; n = 5; corresponding data for loxapine (n = 5) are in Figure S11. *P < 0.05, significantly different as indicated; one‐way ANOVA followed by Tukey's multiple comparisons test. (g) Best consensus score binding mode of CLZ in comparison with the bicuculline‐bound 6X3S structure (Figure S12). Blue arrows point to the positive ionizable feature

The structure‐based pharmacophore screening suggested the bicuculline site as a candidate for all seven compounds (Figures 3c and S11a). In order to investigate whether clozapine or chlorpromazine inhibition in the recombinant α5β3γ2 receptors might be elicited by orthosteric competition, we compared their inhibition at GABA ~EC_5_ and ~EC_20_ (Figure 6a–d). This comparison is indicative of a right‐ward shift for clozapine and thus with a partly or fully competitive mode of action for clozapine. On the other hand, and in line with an allosteric effect, there is no significant change in pIC_50_ values for chlorpromazine between GABA ~EC_5_ and ~EC_20_ (Figure 6c,d).

To further investigate potential use of the orthosteric site, displacement of [^3^H]muscimol by clozapine and chlorpromazine, in direct comparison with bicuculline was performed in hippocampal membranes from rat brain (Figure 6e). The hippocampus contains a high fraction of α5‐containing receptors (Pirker et al., 2000; Sperk et al., 1997). Near complete displacement by bicuculline was observed, as expected (Figure 6e). As has been observed previously in cerebellar and forebrain membranes (Korpi et al., 1995), clozapine incompletely displaces the radioligand. At 1 mM, we see 46% displacement by clozapine and none by chlorpromazine (Figure 6f). Similar experiments with loxapine (1 mM) elicited 71% displacement of [^3^H]muscimol (Figure S11). The lack of displacement by chlorpromazine confirms that it does not inhibit currents via the orthosteric site of α5β3γ2 receptors. In contrast, the displacement by clozapine further supports an orthosteric inhibition, as suggested by the GABA concentration‐dependent degree of current inhibition. Computational docking of clozapine results in a top ranked candidate binding mode, which features the positive ionizable group in the same region of the bicuculline binding site as is observed for the bicuculline‐bound β2/α1 interface (Figures 6g and S12). Thus, the accumulated evidence from the functional and mutational data, the muscimol displacement experiments, pharmacophore screening and computational docking indicate that clozapine inhibits GABA currents in α5β3γ2 receptors by orthosteric inhibition, while chlorpromazine elicits a similar degree of current inhibition by an allosteric mechanism, which is fully or partly mediated by a novel intrasubunit pocket.

## 4 DISCUSSION

Antipsychotic drugs exert functional inhibition of GABA_A_ receptors, with clozapine being the most studied compound, in this regard. Early studies noted incomplete displacement of [^3^H]muscimol and a partly biphasic modulation of [^35^S]TBPS binding (Korpi et al., 1995; Squires & Saederup, 1998), pointing to a complex mode of action. This was further corroborated by additivity studies, in which clozapine was co‐applied with other antipsychotics (Squires & Saederup, 1998). Among the drugs tested together with clozapine were loxapine and clotiapine, both of which had a significantly additive effect when co‐applied with clozapine, compared to the effect of clozapine alone. This is suggesting action on either distinctive subtypes, or different binding sites. These historical studies were methodologically heterogeneous, and subtype‐specific data remained scarce (Squires & Saederup, 1988, 1991, 1993, 1997, 1998, 1999, 2000).

Hippocampal α5‐containing GABA_A_ receptors are considered an emerging target for the treatment of cognitive dysfunction in schizophrenia and other neuropsychiatric conditions (Xu & Wong, 2018), prompting us to investigate clozapine and related compounds in this subtype. The tested compounds exerted inhibitory effects on the actions of sub‐saturating concentrations of GABA on α5β3γ2 receptors, with 100 μM test compound eliciting current reductions ranging from −21% (imipramine) to −85% (loxapine and clotiapine). Interactions of all compounds with GABA_A_ receptors, with the exception of levomepromazine, were previously noted, but the binding sites remained ill‐defined (Asproni et al., 2002; Besnard et al., 2012; Korpi et al., 1995; Michel & Trudeau, 2000; Squires & Saederup, 1988, 1998). Available structural data combined with our computational analysis has now suggested possible involvement of the orthosteric site, and a novel allosteric site which has been described as a chlorpromazine site in the ECD of ELIC (Nys et al., 2016). We thus employed mutational analysis to probe the existence of an intrasubunit ‘chlorpromazine pocket’ in α5 subunits suggested by homology with ELIC (Nys et al., 2016). In total, we find the pharmacophore group comprising chlorpromazine, levomepromazine, nortriptyline and imipramine to be responsive to the introduced mutations in the pocket and, for chlorpromazine, right‐ward shifts were observed. Clozapine, loxapine and clotiapine were not affected by the mutations in the receptor. These findings together suggest that the employed mutants are a specific probe and that the site is likely to exist. However, mutagenesis in a protein region shared by two non‐overlapping binding sites (Figure S6) is liable to be inconclusive. Further studies with direct structural methods thus seem warranted to further clarify how these binding sites are used by chlorpromazine and other related molecules, as mutational analysis cannot serve as definite proof.

We then complemented our functional study with radioligand displacement experiments in hippocampal membranes. At 1 mM, chlorpromazine failed to displace the standard GABA site ligand [^3^H]muscimol, while loxapine and clozapine displaced 71% and 46%, respectively (Figures 6f and S11). For clozapine, the combination of functional and displacement data is fully consistent with orthosteric inhibition of α5β3γ2 receptors. Cumulative evidence from the pharmacophore models and the experimental data suggests that this is also the case for clotiapine and loxapine. Thus, the ligands we investigated here fall into two distinct groups, one acting as orthosteric antagonists, and the other as allosteric negative modulators. In contrast to bicuculline, clozapine appears to be a rather selective orthosteric antagonist that interacts with specific subtypes only (see Figure S6 for subtype differences in the orthosteric pocket) (Rahman et al., 2006).

The body of functional data we present here is intriguingly consistent with all previous data which points to a multiplicity of partly orthosteric and partly allosteric binding sites that are used differentially by molecules with tricyclic cores. We observed no functional inhibition by imipramine and nortriptyline of the highly abundant receptor subtype (α1β3γ2; Figure 2d). The studies by Squires and Saederup did not examine current modulation, but the modulation of GABA inhibition of [^35^S]TBPS binding (Squires & Saederup, 1998), which is a very sensitive indicator for interactions with ortho‐ and allosteric binding sites. Their work also found imipramine and nortriptyline to be almost inactive (Squires & Saederup, 1988). We and others (Korpi et al., 1995; Squires & Saederup, 1998) observed that clozapine displaced [^3^H]muscimol in different brain regions to variable degrees, but never completely, indicative of orthosteric binding only at some subtypes. The additive effects in [^35^S]TBPS modulation (Squires & Saederup, 1998, 2000) and the biphasic effects in the study by Korpi et al are highly indicative of an allosteric component. Our data strongly suggests an orthosteric inhibition in α5β3‐containing GABA_A_ receptors, while the lack of inhibition in α3β3 receptors suggests an unusual subtype dependency. Further studies will be needed to determine precisely the binding sites and net effects of such molecules in individual subtypes to disentangle their potential contributions to both wanted and unwanted effects mediated by GABA_A_ receptors.

There is a long standing debate over whether clozapine exerts part of its therapeutic effects by a GABA‐ergic mechanism of action. Plasma concentrations of clozapine can reach 3 to 4 μM in patients with schizophrenia (Squires & Saederup, 1997; Yada et al., 2021). Consistent with results showing that the elimination half‐life of antipsychotics is several times greater in the human brain than in plasma (Tauscher et al., 2002), a study in rats shows that the concentration of clozapine can be 24‐fold higher in the brain than in the plasma (Squires & Saederup, 1997). Therefore, the therapeutic concentrations of clozapine in the brain can be in the high micromolar range, which would make the concentrations used in this study physiologically relevant. For clozapine and many other antipsychotics, high doses are needed to produce a therapeutic effect (Huhn et al., 2019; Squires & Saederup, 1997). It was already questioned by Squires and Saederup in the 90s (Squires & Saederup, 1997) if these high doses are consistent with their antipsychotic effects, mediated by dopamine or 5‐HT receptors, for which the Ki values are in the low nanomolar range (Seeman, 2006).

Converging evidence points to pivotal alterations of GABA‐ergic signalling in schizophrenia (Charych et al., 2009; Marques et al., 2020; Xu & Wong, 2018), and the effectiveness of the benzodiazepine site ligand bretazenil as antipsychotic monotherapy (Delini‐Stula & Berdah‐Tordjman, 1996) can be interpreted as historical support for the notion that antipsychotic action can be mediated by GABA_A_ receptors. The role of hippocampal α5 subunits in several aspects of memory and cognitive performance has led to the development and subsequent clinical trial of basmisanil. This compound, previously known as RG‐1662 or RO5186582, is an allosteric negative modulator of α5βγ2 receptors and has been under evaluation as an adjunctive therapy in a schizophrenic cohort for the treatment of cognitive impairment associated with schizophrenia. Very intriguingly, the functional inhibition of α5‐containing receptors we observed is much stronger for the antipsychotic compounds (clozapine, loxapine and clotiapine) and relatively weak for the antidepressants nortriptyline and imipramine, or levomepromazine (Figure 2d), which in spite of its canonical classification, does not act as an antipsychotic drug (Huhn et al., 2019).

Accumulated evidence suggests a complex and probably multicausal, aetiology of the pathogenic mechanisms that drive schizophrenia symptoms, involving several neurotransmitter systems including dopamine, GABA and glutamate (Charych et al., 2009). The question thus might not be whether the GABA‐ergic or the dopaminergic system should best be targeted to treat schizophrenia symptoms, but which components of multiple transmitter systems should be targeted in combination for the best results. This is reflected by the notion to combine standard antipsychotic therapy with GABA‐ergic ‘cognition enhancers’, and could potentially be accomplished by compounds with an appropriate polypharmacological profile. Antipsychotic drugs and also many antidepressants display very pronounced polypharmacology. Existing data on our seven tested compounds as reflected in DrugCentral is summarized in Figure S13. In terms of their clinical use, the seven compounds can be grouped into the antipsychotics loxapine, clotiapine, clozapine and chlorpromazine. Levomepromazine, although considered an antipsychotic, is mainly used for its strong sedative effects, while imipramine and nortriptyline are tricyclic antidepressants. In line with the high similarity among these compounds in chemical space, their molecular target profiles overlap broadly with no clear signature that would set the antidepressants apart from the antipsychotics. While still limited to seven compounds, this study suggests that effects at specific GABA_A_ receptor isoforms might separate these two drug classes.

In conclusion, existing evidence suggests a ‘therapeutic portfolio’ mode of action of antipsychotic medications. The exact configuration of an antipsychotic target portfolio remains to be elucidated and is likely to contain both metabotropic and ionotropic receptors (Figure S13 and Tables S8, S9). Hippocampal α5‐containing GABA_A_ receptors are strong candidates, and strongly inhibited by the antipsychotics we tested. Molecules which hit ‘classical’ targets such as D_2_ receptors and GABA_A_ receptors may thus be an attractive alternative to the strategy that drove the development of basmisanil, namely, to augment antipsychotics with GABA‐ergics. Our observation that the degree of functional inhibition we observe in vitro appears to correlate with antipsychotic efficacy is very exciting, but definitely requires systematic investigation with a larger number of compounds and with additional methods in order to substantiate a link between their GABA_A_ receptor effects and their therapeutic benefits. The findings of this study further emphasize the need to identify and characterize allosteric sites which may potentially be targeted and prove useful to avoid the toxicological effects associated with the orthosteric site.

## AUTHOR CONTRIBUTIONS

K.B., M.W., and M.E. conceived the study. K.B. planned and supervised electrophysiological experiments. K.B., L.S., S.R., F.D.V., and F.Z. performed electrophysiological measurements and data analysis. P.S. performed radioligand assays and data analysis. F.K. performed structural analysis and computational docking. T.S., A.G., and T.L. performed similarity analysis, pharmacophore modelling and screening. K.B., M.E., and M.W. wrote the manuscript. K.B., F.K., and F.Z. prepared figures.

## CONFLICT OF INTERESTS

The authors declare no competing interests.

## DECLARATION OF TRANSPARENCY AND SCIENTIFIC RIGOUR

This Declaration acknowledges that this paper adheres to the principles for transparent reporting and scientific rigour of preclinical research, as stated in the BJP guidelines for Design & Analysis and as recommended by funding agencies, publishers and other organisations engaged with supporting research.

## Supporting information




**Data S1.** Supporting Information

## Data Availability

The datasets generated and/or analysed during the current study are available from the corresponding author upon request.
