# Supplementary material for: Tricyclic antipsychotics and antidepressants can inhibit α5‐containing GABAA receptors by two distinct mechanisms
Source: Br J Pharmacol. 2022 Mar 7;179(14):3675–92. doi: 10.1111/bph.15807 (PMC9314015; doi:10.1111/bph.15807)
Supplement: Supplementary file 1 — Data S1. Supporting Information [file BPH-179-3675-s001.pdf]

Supplementary Information

Tricyclic antipsychotics and antidepressants can inhibit α5-containing $GABA_A$ receptors by two distinct mechanisms

Konstantina Bampali (1), Filip Koniuszewski (1), Luca L. Silva (1), Sabah Rehman (2), Florian D. Vogel (1), Thomas Seidel (3), Petra Scholze (1), Florian Zirpel (1), Arthur Garon (3), Thierry Langer (3), Matthäus Willeit (4), Margot Ernst* (1)

(1) Department of Pathobiology of the Nervous System, Center for Brain Research, Medical University Vienna, Spitalgasse 4, 1090 Vienna, Austria

(2) Department of Molecular Neurosciences, Center for Brain Research, Medical University of Vienna, Spitalgasse 4, 1090 Vienna, Austria

(3) Department of Pharmaceutical Sciences, Division of Pharmaceutical Chemistry, University of Vienna, Althanstraße 14, 1090 Vienna, Austria

(4) Department of Psychiatry and Psychotherapy, Medical University of Vienna, Währinger Gürtel 18-20, 1090, Vienna, Austria

*Corresponding author: margot.ernst@meduniwien.ac.at, phone number: +43 1 40160 34065

Supplementary Figure S1

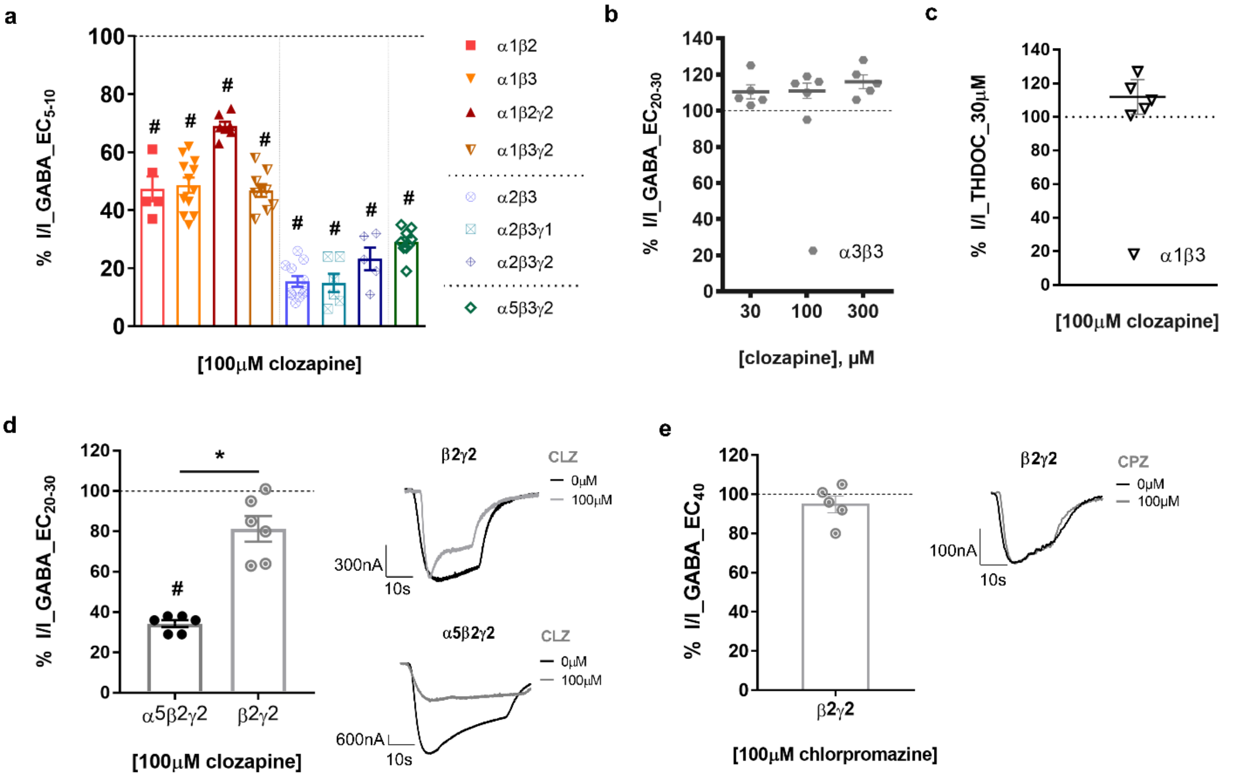

(**a**) 100μM CLZ modulation of currents elicited by an EC_{5-10} GABA concentration in α1β2, α1β2γ2, α1β3, α1β3γ2, α2β3, α2β3γ1, α2β3γ2 and α5β3γ2 receptors. Precise n numbers are in Supplementary Table S1. Statistically significant differences were determined between the receptor subtypes by one-way ANOVA followed by Dunnett's multiple comparisons test, where *p<0.05. All α1-containing receptors were statistically different from α2- and α5-containing receptors. Additionally, one sample t test was performed to determine statistical significance of each mean response from control current and corrected for multiple comparisons using the false discovery rate method of Benjamini and Hochberg, with a discovery rate of 0.05 ($^{\#}p<0.05$). All responses were significantly different from control. (**b**) 30, 100, 300μM CLZ modulation of currents elicited by an EC_{20-30} GABA concentration in α3β3 receptors (n=5). Data are depicted as mean ± SEM. The dotted line is used to visualize the baseline (100%) of control current. (**c**) Modulation of currents elicited by 30μM concentration of the neurosteroid THDOC in α1β3 receptors co-applied with 100μM CLZ, after pre-application of CLZ (n=5). (**d**) Modulation of currents elicited by an EC_{20-30} GABA concentration by 100μM CLZ in α5β2γ2 (n=5) and β2γ2 (n=5) receptors. Columns for each receptor subtype depict mean ± SEM. Statistically significant differences were determined by two-tailed student's t-test, where *p<0.05. One sample t test was performed to determine statistical significance of each mean response from control current, where $^{\#}p<0.05$. The response in β2γ2 receptors was not significantly different from control. Representative traces from electrophysiological recordings of CLZ co-applied with GABA in β2γ2 and α5β2γ2 receptors are also depicted. (**e**) Modulation of currents elicited by an EC_{40} GABA concentration by 100μM in β2γ2 (n=5) receptors. One sample t test was performed to determine statistical significance of each mean response from control current, where $^{\#}p<0.05$. The response in β2γ2 receptors was not significantly different from control.

Supplementary Figure S2

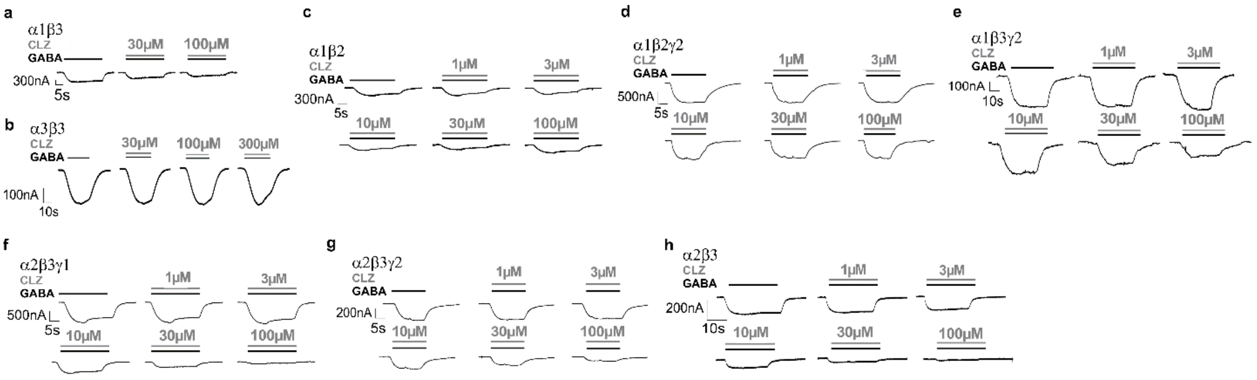

(**a-h**) Representative traces from electrophysiological recordings of CLZ co-applied with GABA in α1β3, α3β3, α1β2, α1β2γ2, α1β3γ2, α2β3γ1, α2β3γ2 and α2β3 receptors. Dose response curves are depicted in Figure 1a.

Supplementary Table S1

Clozapine (CLZ)	α1β2	α1β2γ2	α1β3γ2	α2β3	α2β3γ2	α2β3γ1
IC_{50} (M)	3.36e-005	6.43e-006	9.02e-005	1.64e-005	2.24e-005	1.01e-005
LogIC_{50} ± SEM	-4.47 ± 0.392	-5.19 ± 0.254	-4.04 ± 0.215	-4.133 ± 0.159	-4.65 ± 0.136	-4.99 ± 0.129
nH	= 1	= 1	= 1	= 1	= 1	= 1
% max. efficacy (at 100μM)	46.3	69.1	46.8	15.5	23.2	15.0
n	5	5-7	6-9	5-11	5	6

IC_{50}, LogIC_{50}, Hill slope (nH) and maximum efficacy of clozapine (CLZ) in α1β2, α1β2γ2, α1β3γ2, α2β3, α2β3γ2 and α2β3γ1 receptors, corresponding to the dose response curves depicted in Figure 1a. Control current = 100% (GABA EC_{5-10}). GABA concentrations can be found in Supplementary Table S10.

Supplementary Table S2

Clozapine (CLZ)	$\alpha5\beta3$ (GABA at EC_{20-30})	$\alpha5\beta3\gamma2$ (GABA at EC_{20-30})	$\alpha5\beta3\gamma2$ (GABA at EC_{5-10})
IC_{50} (M)	0.000282	9.35e-005	4.27e-005
LogIC_{50} ± SEM	-3.55 ± 0.0622	-4.03 ± 0.0555	-4.37 ± 0.0334
nH ± SEM	-1.17 ± 0.263	-0.853 ± 0.105	-1 ± 0.0645
% max. efficacy (at 300µM)	45.9	27.5	16.2
n	5-6	5-11	5

IC_{50}, LogIC_{50}, Hill slope (nH) and maximum efficacy of clozapine (CLZ) in $\alpha5\beta3$, $\alpha5\beta3\gamma2$ and $\alpha5\beta3\gamma2$ receptors, corresponding to the dose response curves depicted in Figure 1b and 6a. Control current = 100% (GABA EC_{20-30} and EC_{5-10}). GABA concentrations can be found in Supplementary Table S10.

Supplementary Table S3

Chlorpromazine (CPZ)	$\alpha5\beta3$ (GABA at EC_{20-30})	$\alpha5\beta3\gamma2$ (GABA at EC_{20-30})	$\alpha5\beta3\gamma2$ (GABA at EC_{5-10})
IC_{50} (M)	0.000225	0.000106	0.000121
LogIC_{50} ± SEM	-3.65 ± 0.0368	-3.97 ± 0.0675	-3.92 ± 0.0584
nH ± SEM	-1.46 ± 0.197	-1.1 ± 0.206	-1 ± 0.143
% max. efficacy (at 300µM)	40.0	29.5	32.2
n	6	7-13	5

IC_{50}, LogIC_{50}, Hill slope (nH) and maximum efficacy of chlorpromazine (CPZ) in $\alpha5\beta3$, $\alpha5\beta3\gamma2$ and $\alpha5\beta3\gamma2$ receptors, corresponding to the dose response curves depicted in Figure 1e and 6c. Control current = 100% (GABA EC_{20-30} and EC_{5-10}). GABA concentrations can be found in Supplementary Table S10.

Supplementary Table S4

Drugs	CPZ	IMI	NOR	LEVO	CLOT	LOX	CLZ
CPZ	2	1.874	1.603	1.62	1.292	1.258	1.250
IMI	1.875	2	1.743	1.525	1.318	1.230	1.130
NOR	1.601	1.739	2	1.359	1.283	1.194	1.111
LEVO	1.620	1.523	1.361	2	1.129	1.115	1.161
CLOT	1.292	1.317	1.284	1.129	2	1.820	1.342
LOX	1.257	1.227	1.196	1.116	1.818	2	1.371
CLZ	1.250	1.130	1.113	1.162	1.341	1.373	2
**Tanimoto Combo**							

Drugs	CPZ	IMI	NOR	LEVO	CLOT	LOX	CLZ
CPZ	1	0.932	0.866	0.955	0.766	0.807	0.829
IMI	0.932	1	0.950	0.891	0.819	0.802	0.932
NOR	0.867	0.947	1	0.833	0.829	0.836	0.867
LEVO	0.954	0.89	0.834	1	0.732	0.755	0.954
CLOT	0.766	0.818	0.830	0.724	1	0.970	0.766
LOX	0.806	0.800	0.838	0.755	0.968	1	0.806
CLZ	0.829	0.749	0.762	0.809	0.787	0.835	1
**Shape Tanimoto**							

Drugs	CPZ	IMI	NOR	LEVO	CLOT	LOX	CLZ
CPZ	1	0.942	0.737	0.666	0.526	0.450	0.422
IMI	0.943	1	0.793	0.634	0.499	0.428	0.381
NOR	0.735	0.793	1	0.527	0.454	0.357	0.351
LEVO	0.666	0.634	0.527	1	0.397	0.360	0.353
CLOT	0.526	0.499	0.454	0.405	1	0.849	0.554
LOX	0.450	0.428	0.357	0.360	0.849	1	0.538
CLZ	0.422	0.380	0.351	0.353	0.554	0.538	1
**Color Tanimoto**							

Tables providing the individual shape, color and combo scores. The shape score represents the volumetric overlap of the Van der Waals volumes of the atoms in a ligand pair. Color scores represent the overlap of chemical features. The scores were calculated using the software ROCS and have values ranging from 0 to 1 (2 for Tanimoto Combo) where 0 means no overlap at all and 1 (2 for Tanimoto Combo) means a perfect match. The compound groups that result from proximity in the 2D scatter-plots are emphasized in the combo score table by different background colors.

Supplementary Figure S3

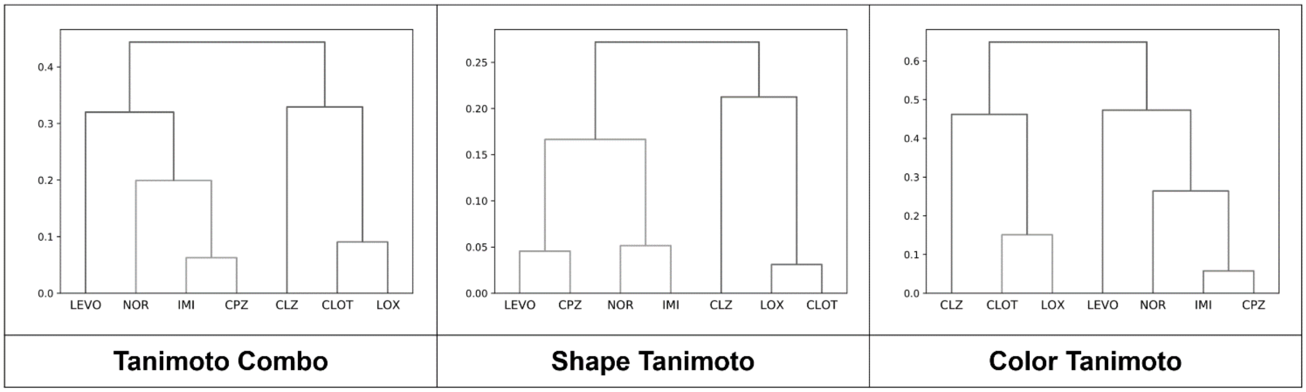

Hierarchical clustering dendrograms for the calculated combo (left), shape (middle) and color Tanimoto similarity scores. The y-axis represents a distance D which was calculated from the similarity scores S as D = 1 – S. The combo score, which ranges from 0 to 2, has been normalized before distance calculation by a factor of 0.5.

Supplementary Figure S4

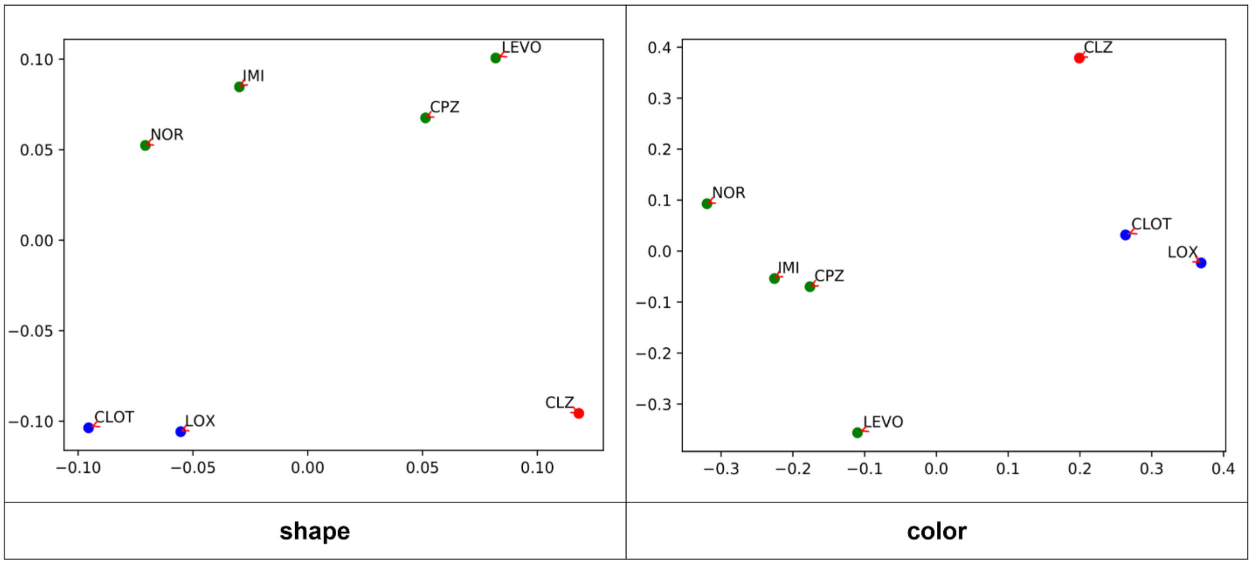

2D scatter-plots reflecting compound similarity in terms of shape (left) and common chemical features (right). The euclidean distance between two compounds correlates with the deviation of their similarity score from 1.0 (= the maximum achievable similarity score). Points are color coded by their membership in the clusters identified from the combo score scatter-plot (Figure 2b).

Supplementary Figure S5

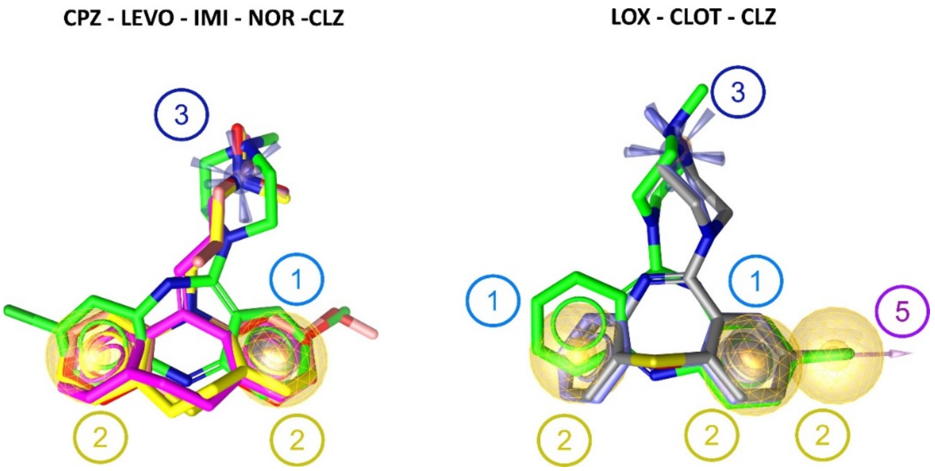

Pharmacophore features of the groups that emerged from the combo score scatter plot in Figure 2b, with CLZ added to each cluster. Features: 1 – aromatic (blue donuts), 2 – hydrophobic (yellow spheres), 3 – positive ionizable (blue stars/rays), 4 – hydrogen bond acceptor (red sphere), 5 – halogen bond (magenta arrow). CLZ can be seen to differ in shape from both groups.

Supplementary Figure S6

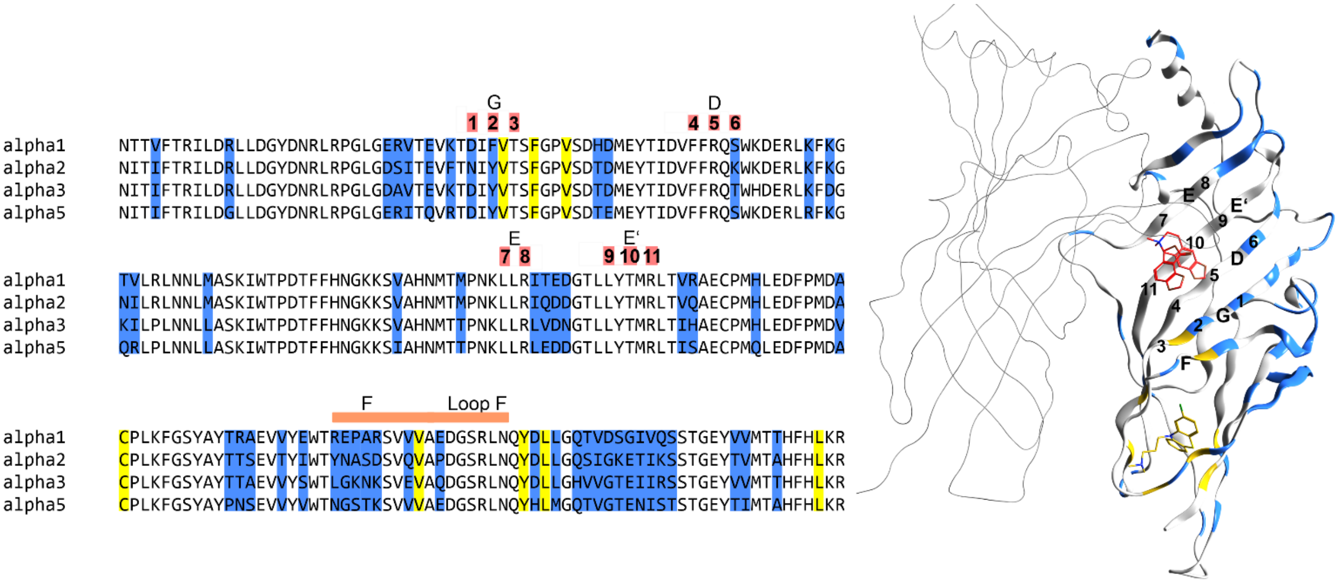

Sequence alignment and the bicuculline bound 6X3S ribbon structure of the ECD. The alignment and the $\alpha1$ subunit are color coded to indicate diverging positions among $\alpha1$, $\alpha2$, $\alpha3$, and $\alpha5$ subunits (blue) in the context of the orthosteric site and the putative intrasubunit CPZ site. The segments (loops) D, E, F and G which contribute to the orthosteric site are labelled, the residues that form the CPZ site are shown in yellow. Strands 1 and 8 (located on segments G and F) contribute to the complementary face of the orthosteric site with sidechains facing towards the interface, and to the intrasubunit CPZ pocket with sidechains facing to the packing core as evidenced by the blue/ yellow alternating colors. Thus, these segments may contribute to potency and efficacy of events at both sites.

Supplementary Table S5

GABA	α5β3γ2	α5L196Wβ3γ2	α5L222Wβ3γ2	α5S189Wβ3γ2	α5F53Wβ3γ2	α5F53W;L222Wβ3γ2
**EC_{50} (M)**	1.35e-005	0.000166	8.32e-006	9.43e-005	6.3e-006	2.15e-005
**$LogEC_{50}$ ± SEM**	-4.87 ± 0.0458	-3.78 ± 0.0336	-5.08 ± 0.099	-4.03 ± 0.0429	-5.2 ± 0.0699	-4.67 ± 0.037
**nH ± SEM**	1.04 ± 0.113	1.18 ± 0.106	0.774 ± 0.143	1.29 ± 0.151	1.07 ± 0.17	1.04 ± 0.09
**amplitude at 1mM (μA)**	4.2 ± 0.8483	2.4 ± 1.06	3.5 ± 0.3056	5.7 ± 0.8419	8.5 ± 0.9414	2.7 ± 0.3489
**n**	5	6	6	6	6	5

EC_{50}, $LogEC_{50}$, Hill slope (nH) and maximum amplitude of 1mM GABA (in μA) in α5β3γ2, α5F53Wβ3γ2, α5S189Wβ3γ2, α5L196Wβ3γ2, α5L222Wβ3γ2 and α5F53W;L222Wβ3γ2 receptors, corresponding to the dose response curves depicted in Figure 4c.

Supplementary Figure S8

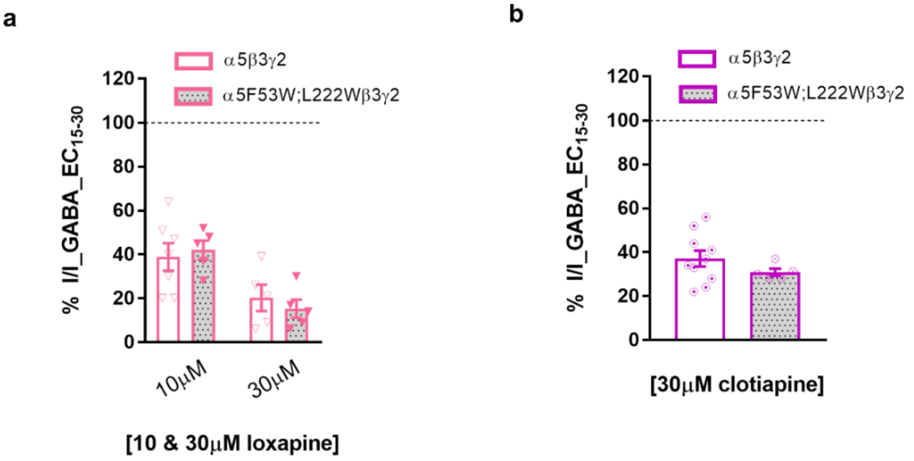

Modulation of currents elicited by an EC_{15-30} GABA concentration by 10 & 30μM LOX (**a**) and 30μM CLOT (**b**) in α5β3γ2 (n=5 for 30μM LOX and n=6 for 10μM LOX, n=10 for 30μM CLOT) and α5F53W;L222Wβ3γ2 (n=5 for 30μM LOX and n=7 for 10μM LOX, n=5 for 30μM CLOT) receptors. Columns for each receptor subtype depict mean ± SEM. Statistically significant differences were determined by two-tailed students t-test, where *$p<0.05$. All responses were found to not be significantly different between wild-type and mutated receptors. The dotted line is used to visualize the baseline (100%) of control current.

Supplementary Figure S9

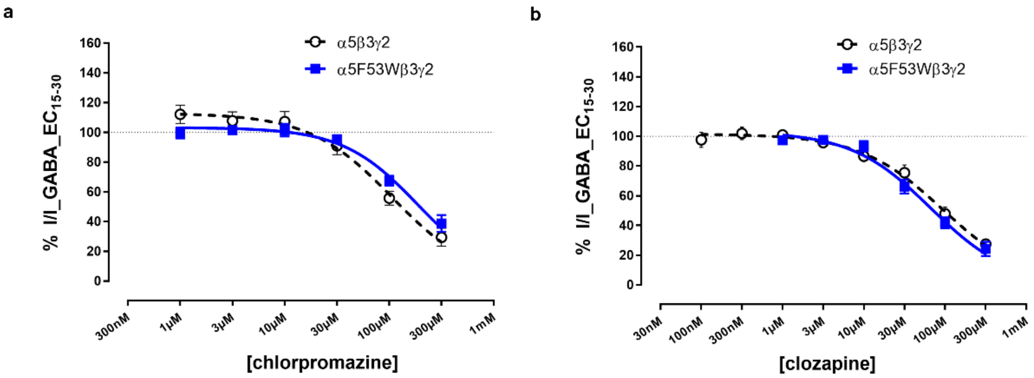

(**a, b**) CLZ (**a**) and CPZ (**d**) dose response curves in α5β3γ2 and α5F53Wβ3γ2 receptors. Data were normalized and fitted to the Hill equation using non-linear regression (fixed bottom of 0) and points are depicted as mean ± SEM. The precise n numbers, as well as the IC_{50}, $logIC_{50}$, Hill slope and maximum efficacy values can be found in Supplementary Tables S6 and S7. Dose response curves in α5β3γ2 receptors are represented with dotted lines, as they are reproduced here for easier comparisons.

Supplementary Table S6

Clozapine (CLZ)	α5β3γ2	α5F53Wβ3γ2	α5L222Wβ3γ2	α5F53W;L222Wβ3γ2
IC_{50} (M)	9.35e-005	6.69e-005	7.16e-005	8.81e-005
$LogIC_{50}$ ± SEM	-4.03 ± 0.0555	-4.17 ± 0.0777	-4.15 ± 0.076	-4.06 ± 0.0674
nH ± SEM	-0.853 ± 0.105	-0.886 ± 0.145	-0.753 ± 0.114	-0.749 ± 0.108
% max. efficacy (at 300μM)	27.5	24.2	30.0	32.3
n	5-11	5-11	5-11	5-11

IC_{50}, $LogIC_{50}$, Hill slope (nH) and maximum efficacy of clozapine (CLZ) in α5β3γ2, α5F53Wβ3γ2, α5L222Wβ3γ2 and α5F53W;L222Wβ3γ2 receptors, corresponding to the dose response curves depicted in Figure 5a and in Supplementary Figure S9. Control current = 100% (GABA EC_{20-30}). GABA concentrations can be found in Supplementary Table S10.

Supplementary Table S7

Chlorpromazine (CPZ)	α5β3γ2	α5F53Wβ3γ2	α5L222Wβ3γ2	α5F53W;L222Wβ3γ2
IC_{50} (M)	0.000106	0.000186	0.00035	0.000236
$LogIC_{50}$ ± SEM	-3.97 ± 0.0675	-3.73 ± 0.0467	-3.46 ± 0.0845	-3.63 ± 0.071
nH ± SEM	-1.1 ± 0.206	-1.22 ± 0.176	-1.11 ± 0.268	-0.79 ± 0.145
% max. efficacy (at 300μM)	29.5	38.8	55.9	51.8
n	7-13	5-13	5-11	5-14

IC_{50}, $LogIC_{50}$, Hill slope (nH) and maximum efficacy of chlorpromazine (CPZ) in α5β3γ2, α5F53Wβ3γ2, α5L222Wβ3γ2 and α5F53W;L222Wβ3γ2 receptors, corresponding to the dose response curves depicted in Figure 5d and in Supplementary Figure S9. Control current = 100% (GABA EC_{20-30}). GABA concentrations can be found in Supplementary Table S10.

Supplementary Figure S10

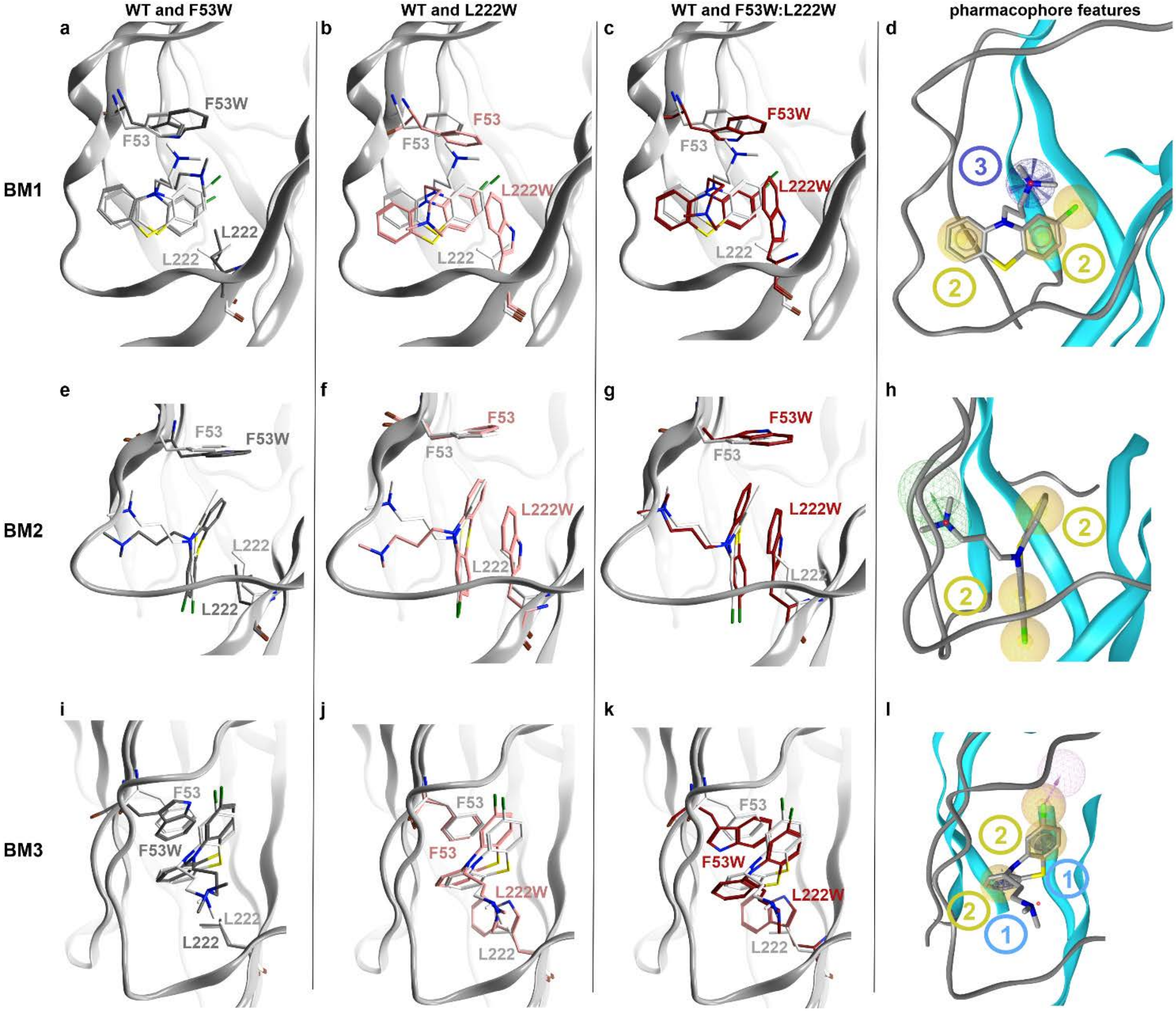

Best CPZ binding modes. CPZ docking into 6A96 WT (white) and the selected mutants F53W (grey), L222W (pink), F53W;L222W (red), showing the three different candidate binding modes (BM1-3) which are consistent with a small right shift (loss of apparent affinity) in L222W and F53W;L222W. Binding mode 3 (panels i-l) shows similarity to the CPZ in the ELIC bound structure. Panels d, h, l display the interacting features as identified by Ligand Scout for the three binding modes. Of note, binding mode 1 is in best agreement with the ligand based analysis (Figure 2c).

Supplementary Figure S11

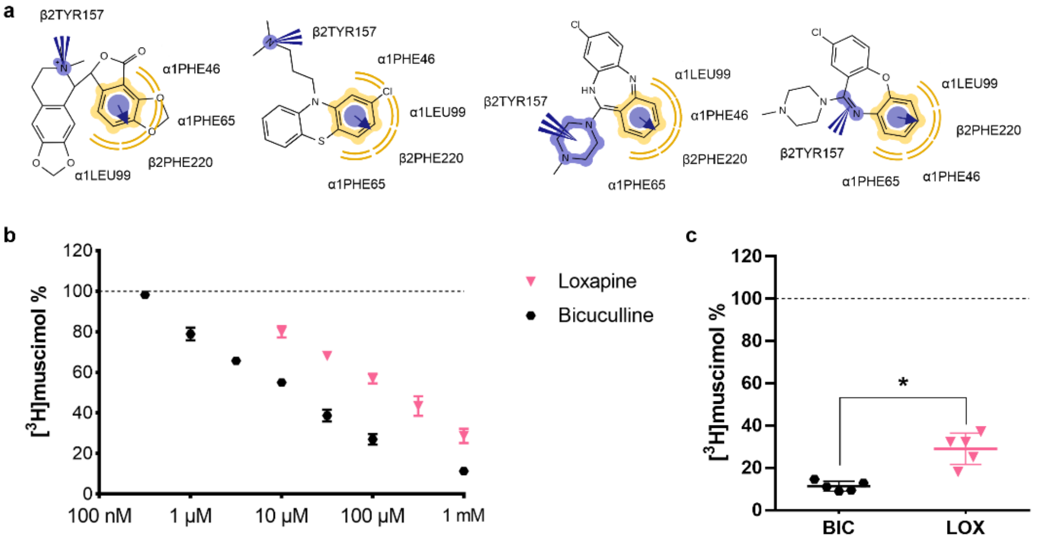

(**a**) 2D representations of BIC, CPZ, CLZ and LOX with the matched features identified in the screen (yellow sphere: hydrophobic, blue donut: aromatic, blue rays: cationic). The cationic feature forms cation-pi interactions with Y157, the hydrophobic/aromatic feature is in close contact with multiple aromatic and hydrophobic sidechains. (**b**) Inhibition of binding of [3H]muscimol to rat hippocampal membrane $GABA_A$ receptors (n=3-5). Membranes were incubated with 10nM [3H]muscimol in the presence of various concentrations of the displacing ligand. 100% is the amount of radioligand bound in the presence of 1% DMSO. Data shown are mean ± SEM of three independent experiments performed in duplicates each (for the concentrations <1mM) and five independent experiments performed in duplicates each (for 1mM). Visual inspection and sigmoid fitting indicated that the displacement points are not described by a single sigmoid function, as would be expected due to different affinities for the diversity of subtypes that are present in hippocampal membranes. Therefore, the individual points are displayed without fitting. (**c**) Inhibition of binding of [3H]muscimol to rat hippocampal membrane $GABA_A$ receptors at 1mM BIC and LOX. Hippocampal membranes from five independent membrane preparations were incubated with 10nM [3H]muscimol in the presence of 1mM of displacing ligand in five independent experiments performed in duplicates each. One-way ANOVA followed by Tukey's multiple comparisons test was performed to determine statistically significant differences between BIC (n=5), LOX (n=5), CLZ (n=5) and CPZ (n=5), where *p<0.05 (see Figure 6f).

Supplementary Figure S12

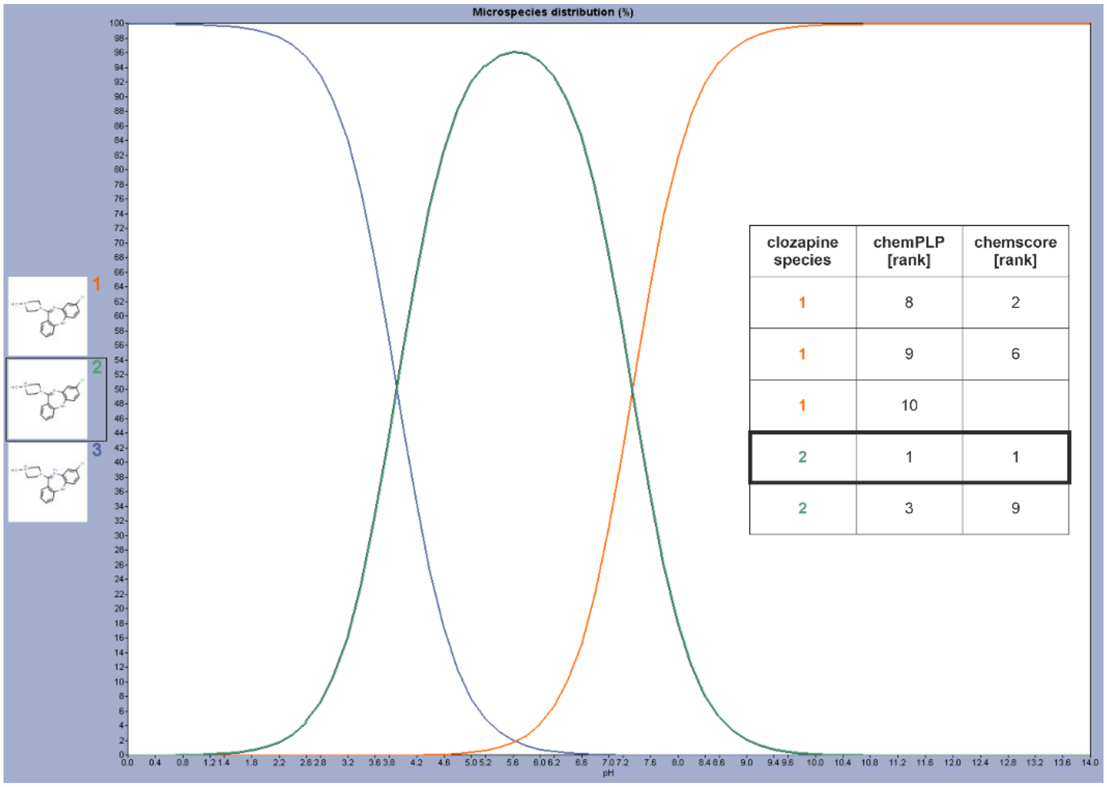

clozapine species	chemPLP [rank]	chemscore [rank]
1	8	2
1	9	6
1	10	
2	1	1
2	3	9

The pKa-function of MarvinSketch was used to select the protonation states of CLZ for the docking based on pKa-predictions using pKa values and microspecies distribution of the nitrogens in CLZ which are susceptible to protonation/deprotonation. The orange curve (1) corresponds to the completely unprotonated species (1), the green curve (2) to the species only protonated at the methyl-substituted piperazine nitrogen, and the blue curve (3) to the species additionally protonated at the amidine nitrogen. Thus, species 1 and 2 were docked as described in the methods. The tabular inset provides the scores from the top 10 solutions of the best candidate binding mode that is displayed in Figure 6g. The protonated species 2 assumes a binding mode which is top ranked with both scoring functions (chemPLP and chemsocore), and a very similar solution is found in two more top ten positions. Interestingly, the unprotonated species 1 can also assume this binding mode which is found a total of five times in the top ten results.

Supplementary Figure S13

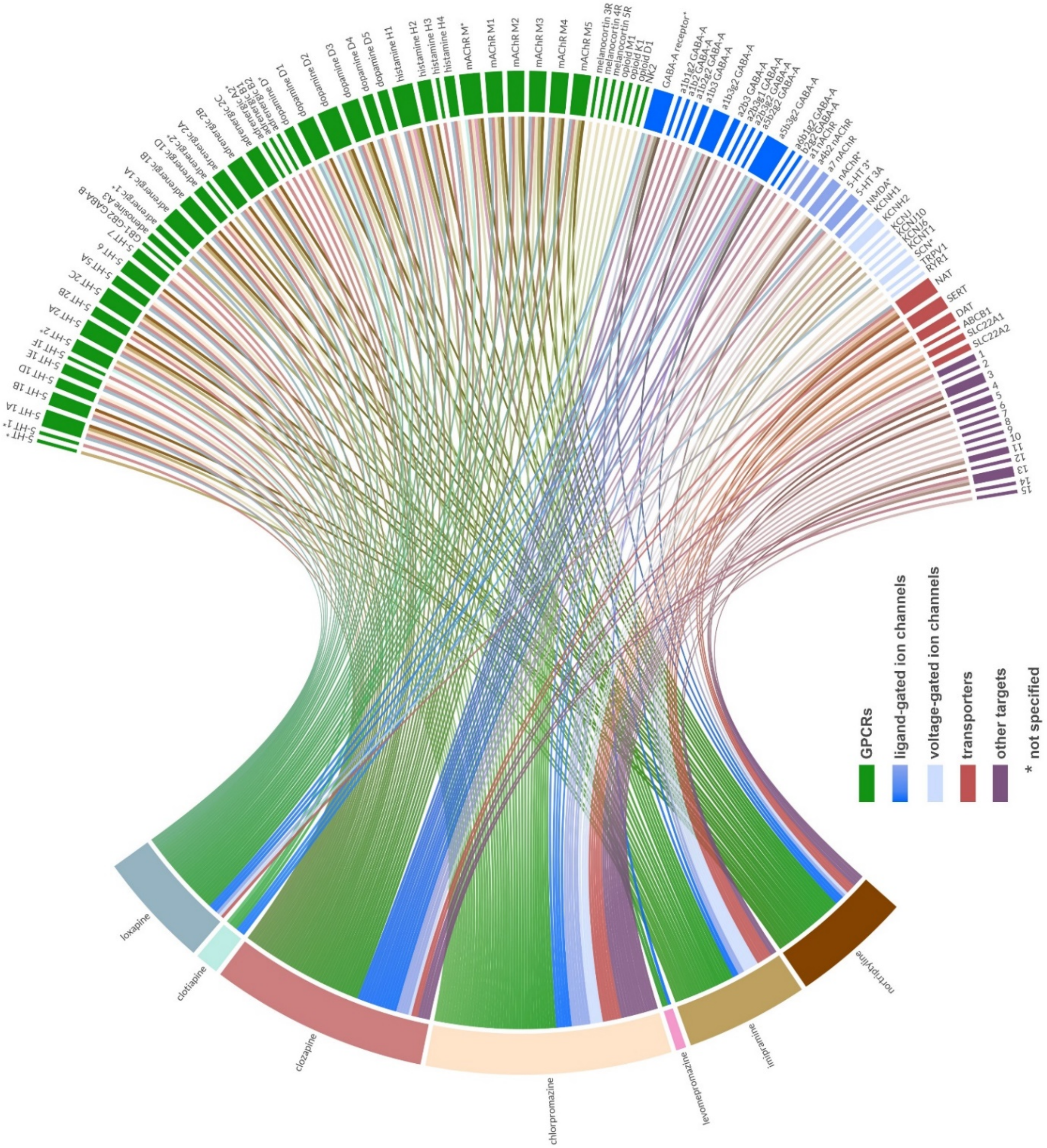

Chord diagram representing the LOX, CLOT, CLZ, CPZ, LEVO, IMI and NOR (left/bottom) and their mammalian drug targets (right/top) grouped according to the IUPHAR recommended categories. "Other targets" are described in more detail in Supplementary Table S8. All drugs and target full names, as well as their sources are listed in Supplementary Table S9. For the chord diagram, DrugCentral (https://drugcentral.org/ accessed on 06.01.2020) was used and only mammalian drug targets were taken into account. Additionally, for $GABA_A$ and $GABA_B$ as well as for nAChRs, literature findings were added (Supplementary Table S9)[1-10]. Terminology was unified across all drug targets (since differences exist, e.g. Serotonin ($5\text{-}HT_3$) receptor 3 and 5-hydroxytryptamine receptor 3). Targets were grouped according to the IUPHAR recommended categories. The chord diagram was created with python 3.8 and the python package chord (https://pypi.org/project/chord/).

Supplementary Table S8

Numbering	Name	Gene name
1	Adenylate cyclase	ADCY*
2	Arachidonate 15-lipoxygenase	ALOX15
3	Aldehyde oxidase	AOX1
4	Cytochrome P450 2C19	CYP2C19
5	Cytochrome P450 2D6	CYP2D6
6	Epidermal growth factor receptor	EGFR
7	Receptor tyrosine-protein kinase erB-2	ERBB2
8	Tyrosine-protein kinase Fyn	FYN
9	FAD-linked sulfhydryl oxidase ALR	GFER
10	Membrane-associated progesterone receptor component 1	PGRMC1
11	Major prion protein	PRNP
12	Proto-oncogene tyrosine-protein kinase receptor Ret	RET
13	Sigma non-opioid intracellular receptor 1	SIGMAR1
14	UDP-glucuronosyltransferase 1-4	UGT1A4
15	Calmodulin	CALM*

Drug target names and their gene names according to the numbering used in the cluster “other targets” in Supplementary Figure S13.

Supplementary Table S9

Drug	Drug target (full name)	Sources
clozapine	Sodium-dependent noradrenaline transporter	DrugCentral
clozapine	Sodium-dependent noradrenaline transporter	DrugCentral
clozapine	Sodium-dependent noradrenaline transporter	DrugCentral
clozapine	Sodium-dependent serotonin transporter	DrugCentral
chlorpromazine	Sodium-dependent noradrenaline transporter	DrugCentral
chlorpromazine	Sodium-dependent dopamine transporter	DrugCentral
chlorpromazine	Multidrug resistance protein 1	DrugCentral
chlorpromazine	Solute carrier family 22 member 1	DrugCentral
chlorpromazine	Sodium-dependent serotonin transporter	DrugCentral
chlorpromazine	Solute carrier family 22 member 2	DrugCentral
loxapine	Sodium-dependent noradrenaline transporter	DrugCentral
imipramine	Sodium-dependent dopamine transporter	DrugCentral
imipramine	Sodium-dependent serotonin transporter	DrugCentral
imipramine	Sodium-dependent dopamine transporter	DrugCentral
imipramine	Sodium-dependent noradrenaline transporter	DrugCentral
imipramine	Solute carrier family 22 member 1	DrugCentral
imipramine	Sodium-dependent noradrenaline transporter	DrugCentral
imipramine	Sodium-dependent serotonin transporter	DrugCentral
imipramine	Sodium-dependent noradrenaline transporter	DrugCentral
imipramine	Sodium-dependent serotonin transporter	DrugCentral
imipramine	Solute carrier family 22 member 2	DrugCentral
nortriptyline	Sodium-dependent dopamine transporter	DrugCentral
nortriptyline	Sodium-dependent noradrenaline transporter	DrugCentral
nortriptyline	Sodium-dependent serotonin transporter	DrugCentral
chlorpromazine	Major prion protein	DrugCentral
imipramine	Major prion protein	DrugCentral
chlorpromazine	Sigma non-opioid intracellular receptor 1	DrugCentral
chlorpromazine	Membrane-associated progesterone receptor component 1	DrugCentral
imipramine	G protein-activated inward rectifier potassium channel 4	DrugCentral
clozapine	Sigma non-opioid intracellular receptor 1	DrugCentral
imipramine	Sigma non-opioid intracellular receptor 1	DrugCentral
clozapine	5-hydroxytryptamine receptor 3A	DrugCentral
clozapine	Potassium voltage-gated channel subfamily H member 2	DrugCentral
clozapine	5-hydroxytryptamine receptor 3A	DrugCentral
clozapine	5-hydroxytryptamine receptor 3*	DrugCentral
clozapine	a1b2 GABA-A	from this study
clozapine	a1b3 GABA-A	from this study

clozapine	a1b1g2 GABA-A	Korpi et al. 1995[6]
clozapine	a1b2g2 GABA-A	from this study, Korpi et al. 1995, Asproni et al. 2002[2,6]
clozapine	a1b3g2 GABA-A	from this study, Korpi et al. 1995[6]
clozapine	a2b3 GABA-A	from this study
clozapine	a2b3g1 GABA-A	from this study
clozapine	a2b3g2 GABA-A	from this study
clozapine	a5b2g2 GABA-A	from this study
clozapine	a5b3g2 GABA-A	from this study
clozapine	a6b1g2 GABA-A	Korpi et al. 1995[6]
clozapine	b2g2 GABA-A	from this study
chlorpromazine	a1b3 GABA-A	from this study
chlorpromazine	a1b3g2 GABA-A	from this study
chlorpromazine	a2b3 GABA-A	from this study
chlorpromazine	a5b3g2 GABA-A	from this study
nortriptyline	a1b3g2 GABA-A	from this study
nortriptyline	a5b3g2 GABA-A	from this study
imipramine	a5b3g2 GABA-A	from this study
levomepromazine	a5b3g2 GABA-A	from this study
loxapine	a1b3g2 GABA-A	from this study
loxapine	a5b3g2 GABA-A	from this study
clotiapine	a1b3g2 GABA-A	from this study
clotiapine	a5b3g2 GABA-A	from this study
nortriptyline	Nicotinic acetylcholine receptor*	Weber et al. 2013[10]
imipramine	Nicotinic acetylcholine receptor*	Weber et al. 2013[10]
clozapine	Nicotinic acetylcholine receptor alpha 7	Singhal et al. 2007[7]
chlorpromazine	Nicotinic acetylcholine receptor alpha 7	Ashoor et al. 2011[1]
clozapine	Nicotinic acetylcholine receptor a4b2	Grinevich et al. 2009[5]
chlorpromazine	Nicotinic acetylcholine receptor a4b2	Grinevich et al. 2009[5]
chlorpromazine	Nicotinic acetylcholine receptor*	Gotti et al. 2006[4]
chlorpromazine	5-hydroxytryptamine receptor 3A	DrugCentral
chlorpromazine	Glutamate NMDA receptor	DrugCentral
chlorpromazine	Sodium channel alpha subunits*	DrugCentral
chlorpromazine	Transient receptor potential cation channel subfamily V member 1	DrugCentral
chlorpromazine	Acetylcholine receptor subunit alpha 1	DrugCentral
chlorpromazine	Glutamate NMDA receptor	DrugCentral
chlorpromazine	Potassium voltage-gated channel subfamily H member 2	DrugCentral
chlorpromazine	Ryanodine receptor 1	DrugCentral
loxapine	5-hydroxytryptamine receptor 3A	DrugCentral
loxapine	Potassium channel subfamily T member 1	DrugCentral

imipramine	Potassium voltage-gated channel subfamily H member 1	DrugCentral
imipramine	Glutamate NMDA receptor	DrugCentral
imipramine	Sodium channel alpha*	DrugCentral
imipramine	Potassium voltage-gated channel subfamily H member 2	DrugCentral
imipramine	G protein-activated inward rectifier potassium channel 2	DrugCentral
nortriptyline	ATP-sensitive inward rectifier potassium channel 10	DrugCentral
clozapine	5-hydroxytryptamine receptor 6	DrugCentral
clozapine	dopamine receptor D(4)	DrugCentral
clozapine	Muscarinic acetylcholine receptor M3	DrugCentral
clozapine	Histamine H1 receptor	DrugCentral
clozapine	dopamine receptor D(1B)	DrugCentral
clozapine	adrenergic receptor Alpha-1D	DrugCentral
clozapine	5-hydroxytryptamine receptor 1F	DrugCentral
clozapine	5-hydroxytryptamine receptor 1E	DrugCentral
clozapine	5-hydroxytryptamine receptor 1D	DrugCentral
clozapine	5-hydroxytryptamine receptor 1B	DrugCentral
clozapine	adrenergic receptor Alpha-1B	DrugCentral
clozapine	dopamine receptor D(3)	DrugCentral
clozapine	adrenergic receptor Alpha-2B	DrugCentral
clozapine	5-hydroxytryptamine receptor 7	DrugCentral
clozapine	5-hydroxytryptamine receptor 2B	DrugCentral
clozapine	5-hydroxytryptamine receptor 2A	DrugCentral
clozapine	dopamine receptor D(2)	DrugCentral
clozapine	dopamine receptor D(1A)	DrugCentral
clozapine	adrenergic receptor Alpha-1B	DrugCentral
clozapine	adrenergic receptor Alpha-2C	DrugCentral
clozapine	Muscarinic acetylcholine receptor M5	DrugCentral
clozapine	Muscarinic acetylcholine receptor M4	DrugCentral
clozapine	Muscarinic acetylcholine receptor M2	DrugCentral
clozapine	Muscarinic acetylcholine receptor M1	DrugCentral
clozapine	Histamine H4 receptor	DrugCentral
clozapine	Histamine H2 receptor	DrugCentral
clozapine	adrenergic receptor Alpha-1A	DrugCentral
clozapine	5-hydroxytryptamine receptor 6	DrugCentral
clozapine	5-hydroxytryptamine receptor 2C	DrugCentral
clozapine	adrenergic receptor Alpha-2A	DrugCentral
clozapine	Adenosine receptor A3	DrugCentral
clozapine	Histamine H1 receptor	DrugCentral
clozapine	Dopamine receptor*	DrugCentral

clozapine	5-hydroxytryptamine receptor 7	DrugCentral
clozapine	dopamine receptor D(1A)	DrugCentral
clozapine	adrenergic receptor Beta-1	DrugCentral
clozapine	dopamine receptor D(4)	DrugCentral
clozapine	Muscarinic acetylcholine receptor*	DrugCentral
clozapine	5-hydroxytryptamine receptor 1A	DrugCentral
clozapine	dopamine receptor D(2)	DrugCentral
clozapine	dopamine receptor D(2)	DrugCentral
clozapine	dopamine receptor D(3)	DrugCentral
clozapine	5-hydroxytryptamine receptor 5A	DrugCentral
clozapine	5-hydroxytryptamine receptor 2*	DrugCentral
clozapine	Dopamine receptor*	DrugCentral
clozapine	adrenergic receptor alpha-2*	DrugCentral
clozapine	adrenergic receptor Alpha-1A	DrugCentral
clozapine	Muscarinic acetylcholine receptor M1	DrugCentral
clozapine	Muscarinic acetylcholine receptor M2	DrugCentral
clozapine	dopamine receptor D(3)	DrugCentral
clozapine	adrenergic receptor Alpha-1A	DrugCentral
clozapine	5-hydroxytryptamine receptor 1*	DrugCentral
clozapine	5-hydroxytryptamine receptor 1B	DrugCentral
clozapine	5-hydroxytryptamine receptor 1A	DrugCentral
clozapine	dopamine receptor D(1A)	DrugCentral
clozapine	adrenergic receptor Alpha-2A	DrugCentral
clozapine	Histamine H1 receptor	DrugCentral
clozapine	dopamine receptor D(2)	DrugCentral
clozapine	5-hydroxytryptamine receptor 2*	DrugCentral
clozapine	5-hydroxytryptamine receptor 2A	DrugCentral
clozapine	dopamine receptor D(2)	DrugCentral
clozapine	dopamine receptor D(1A)	DrugCentral
clozapine	5-hydroxytryptamine receptor 2C	DrugCentral
clozapine	5-hydroxytryptamine receptor 1A	DrugCentral
clozapine	5-hydroxytryptamine receptor 2A	DrugCentral
clozapine	adrenergic receptor Beta-2	DrugCentral
clozapine	adrenergic receptor Beta-1	DrugCentral
clozapine	5-hydroxytryptamine receptor 5A	DrugCentral
clozapine	5-hydroxytryptamine receptor 7	DrugCentral
clozapine	Histamine H4 receptor	DrugCentral
clozapine	Histamine H4 receptor	DrugCentral
clozapine	Histamine H3 receptor	DrugCentral

clozapine	GABA-B	DrugCentral
nortriptyline	Muscarinic acetylcholine receptor*	DrugCentral
loxapine	Muscarinic acetylcholine receptor M1	DrugCentral
levomepromazine	Muscarinic acetylcholine receptor*	DrugCentral
chlorpromazine	adrenergic receptor Alpha-1A	DrugCentral
chlorpromazine	dopamine receptor D(4)	DrugCentral
chlorpromazine	Histamine H4 receptor	DrugCentral
chlorpromazine	Histamine H1 receptor	DrugCentral
chlorpromazine	5-hydroxytryptamine receptor 5A	DrugCentral
chlorpromazine	adrenergic receptor Alpha-1B	DrugCentral
chlorpromazine	5-hydroxytryptamine receptor 1E	DrugCentral
chlorpromazine	5-hydroxytryptamine receptor 1B	DrugCentral
chlorpromazine	5-hydroxytryptamine receptor 1D	DrugCentral
chlorpromazine	Histamine H2 receptor	DrugCentral
chlorpromazine	Muscarinic acetylcholine receptor M3	DrugCentral
chlorpromazine	Muscarinic acetylcholine receptor M1	DrugCentral
chlorpromazine	Muscarinic acetylcholine receptor M5	DrugCentral
chlorpromazine	Muscarinic acetylcholine receptor M4	DrugCentral
chlorpromazine	Muscarinic acetylcholine receptor M2	DrugCentral
chlorpromazine	Mu-type opioid receptor	DrugCentral
chlorpromazine	adrenergic receptor Alpha-1D	DrugCentral
chlorpromazine	Kappa-type opioid receptor	DrugCentral
chlorpromazine	Delta-type opioid receptor	DrugCentral
chlorpromazine	Substance-K receptor	DrugCentral
chlorpromazine	adrenergic receptor Alpha-1B	DrugCentral
chlorpromazine	5-hydroxytryptamine receptor 1B	DrugCentral
chlorpromazine	Melanocortin receptor 4	DrugCentral
chlorpromazine	Melanocortin receptor 5	DrugCentral
chlorpromazine	Melanocortin receptor 3	DrugCentral
chlorpromazine	Dopamine receptor*	DrugCentral
chlorpromazine	5-hydroxytryptamine receptor 7	DrugCentral
chlorpromazine	Muscarinic acetylcholine receptor*	DrugCentral
chlorpromazine	5-hydroxytryptamine receptor 1A	DrugCentral
chlorpromazine	dopamine receptor D(2)	DrugCentral
chlorpromazine	5-hydroxytryptamine receptor 2*	DrugCentral
chlorpromazine	Dopamine receptor*	DrugCentral
chlorpromazine	5-hydroxytryptamine receptor 1A	DrugCentral
chlorpromazine	dopamine receptor D(1A)	DrugCentral
chlorpromazine	dopamine receptor D(2)	DrugCentral

chlorpromazine	5-hydroxytryptamine receptor 2B	DrugCentral
chlorpromazine	dopamine receptor D(3)	DrugCentral
chlorpromazine	5-hydroxytryptamine receptor 2C	DrugCentral
chlorpromazine	5-hydroxytryptamine receptor 2A	DrugCentral
chlorpromazine	dopamine receptor D(2)	DrugCentral
chlorpromazine	5-hydroxytryptamine receptor 6	DrugCentral
chlorpromazine	dopamine receptor D(1A)	DrugCentral
chlorpromazine	adrenergic receptor Alpha-2C	DrugCentral
chlorpromazine	dopamine receptor D(1B)	DrugCentral
chlorpromazine	adrenergic receptor Alpha-2B	DrugCentral
chlorpromazine	5-hydroxytryptamine receptor 7	DrugCentral
chlorpromazine	adrenergic receptor Alpha-2A	DrugCentral
chlorpromazine	Histamine H1 receptor	DrugCentral
chlorpromazine	adrenergic receptor Alpha-1A	DrugCentral
chlorpromazine	5-hydroxytryptamine receptor 6	DrugCentral
chlorpromazine	5-hydroxytryptamine receptor 7	DrugCentral
loxapine	5-hydroxytryptamine receptor 6	DrugCentral
loxapine	Histamine H4 receptor	DrugCentral
loxapine	adrenergic receptor Alpha-1A	DrugCentral
loxapine	5-hydroxytryptamine receptor 5A	DrugCentral
loxapine	adrenergic receptor Alpha-1B	DrugCentral
loxapine	5-hydroxytryptamine receptor 1E	DrugCentral
loxapine	5-hydroxytryptamine receptor 1B	DrugCentral
loxapine	5-hydroxytryptamine receptor 1D	DrugCentral
loxapine	Histamine H2 receptor	DrugCentral
loxapine	dopamine receptor D(1B)	DrugCentral
loxapine	dopamine receptor D(1A)	DrugCentral
loxapine	Muscarinic acetylcholine receptor M3	DrugCentral
loxapine	adrenergic receptor Alpha-2C	DrugCentral
loxapine	adrenergic receptor Alpha-2B	DrugCentral
loxapine	adrenergic receptor Alpha-2A	DrugCentral
loxapine	Muscarinic acetylcholine receptor M5	DrugCentral
loxapine	5-hydroxytryptamine receptor 1A	DrugCentral
loxapine	Muscarinic acetylcholine receptor M4	DrugCentral
loxapine	Muscarinic acetylcholine receptor M2	DrugCentral
loxapine	5-hydroxytryptamine receptor 2C	DrugCentral
loxapine	Histamine H1 receptor	DrugCentral
loxapine	5-hydroxytryptamine receptor 7	DrugCentral
loxapine	Adrenergic receptor alpha-1	DrugCentral

loxapine	Muscarinic acetylcholine receptor M1	DrugCentral
loxapine	5-hydroxytryptamine receptor 6	DrugCentral
loxapine	dopamine receptor D(2)	DrugCentral
loxapine	dopamine receptor D(4)	DrugCentral
loxapine	5-hydroxytryptamine receptor 2A	DrugCentral
loxapine	dopamine receptor D(3)	DrugCentral
loxapine	Histamine H1 receptor	DrugCentral
loxapine	dopamine receptor D(2)	DrugCentral
loxapine	5-hydroxytryptamine receptor 2C	DrugCentral
loxapine	5-hydroxytryptamine receptor 2A	DrugCentral
clotiapine	Muscarinic acetylcholine receptor*	DrugCentral
clotiapine	5-hydroxytryptamine receptor 2*	DrugCentral
clotiapine	dopamine receptor D(1A)	DrugCentral
clotiapine	dopamine receptor D(2)	DrugCentral
clotiapine	dopamine receptor D(2)	DrugCentral
imipramine	5-hydroxytryptamine receptor 1A	DrugCentral
imipramine	5-hydroxytryptamine receptor 2*	DrugCentral
imipramine	Adenosine receptor A3	DrugCentral
imipramine	Dopamine receptor*	DrugCentral
imipramine	Muscarinic acetylcholine receptor M5	DrugCentral
imipramine	adrenergic receptor Alpha-1A	DrugCentral
imipramine	Adrenergic receptor alpha-2	DrugCentral
imipramine	5-hydroxytryptamine receptor 2C	DrugCentral
imipramine	Muscarinic acetylcholine receptor*	DrugCentral
imipramine	Adrenergic receptor alpha-1	DrugCentral
imipramine	dopamine receptor D(2)	DrugCentral
imipramine	Histamine H1 receptor	DrugCentral
imipramine	5-hydroxytryptamine receptor*	DrugCentral
imipramine	Muscarinic acetylcholine receptor M5	DrugCentral
imipramine	Muscarinic acetylcholine receptor M4	DrugCentral
imipramine	Muscarinic acetylcholine receptor M3	DrugCentral
imipramine	Muscarinic acetylcholine receptor M2	DrugCentral
imipramine	Muscarinic acetylcholine receptor M1	DrugCentral
imipramine	Histamine H1 receptor	DrugCentral
imipramine	dopamine receptor D(3)	DrugCentral
imipramine	adrenergic receptor Alpha-2B	DrugCentral
imipramine	adrenergic receptor Alpha-1A	DrugCentral
imipramine	5-hydroxytryptamine receptor 2C	DrugCentral
imipramine	5-hydroxytryptamine receptor 2A	DrugCentral

imipramine	dopamine receptor D(2)	DrugCentral
nortriptyline	dopamine receptor D(1A)	DrugCentral
nortriptyline	adrenergic receptor Alpha-2A	DrugCentral
nortriptyline	5-hydroxytryptamine receptor 2B	DrugCentral
nortriptyline	dopamine receptor D(3)	DrugCentral
nortriptyline	Histamine H2 receptor	DrugCentral
nortriptyline	adrenergic receptor Alpha-2C	DrugCentral
nortriptyline	adrenergic receptor Alpha-1D	DrugCentral
nortriptyline	adrenergic receptor Alpha-1B	DrugCentral
nortriptyline	5-hydroxytryptamine receptor 1B	DrugCentral
nortriptyline	5-hydroxytryptamine receptor 6	DrugCentral
nortriptyline	adrenergic receptor Alpha-1A	DrugCentral
nortriptyline	Histamine H1 receptor	DrugCentral
nortriptyline	Muscarinic acetylcholine receptor M5	DrugCentral
nortriptyline	Muscarinic acetylcholine receptor M4	DrugCentral
nortriptyline	Muscarinic acetylcholine receptor M3	DrugCentral
nortriptyline	Muscarinic acetylcholine receptor M2	DrugCentral
nortriptyline	Muscarinic acetylcholine receptor M1	DrugCentral
nortriptyline	Histamine H1 receptor	DrugCentral
nortriptyline	adrenergic receptor Alpha-2B	DrugCentral
nortriptyline	adrenergic receptor Alpha-1A	DrugCentral
nortriptyline	5-hydroxytryptamine receptor 2C	DrugCentral
nortriptyline	5-hydroxytryptamine receptor 2A	DrugCentral
nortriptyline	5-hydroxytryptamine receptor 1A	DrugCentral
nortriptyline	dopamine receptor D(2)	DrugCentral
nortriptyline	5-hydroxytryptamine receptor 1A	DrugCentral
levomepromazine	Histamine H1 receptor	DrugCentral
clozapine	Aldehyde oxidase	DrugCentral
clozapine	UDP-glucuronosyltransferase 1-4	DrugCentral
clozapine	Adenylate cyclase*	DrugCentral
chlorpromazine	Epidermal growth factor receptor	DrugCentral
chlorpromazine	Tyrosine-protein kinase Fyn	DrugCentral
chlorpromazine	Receptor tyrosine-protein kinase erbB-2	DrugCentral
chlorpromazine	Arachidonate 15-lipoxygenase	DrugCentral
chlorpromazine	Adenylate cyclase*	DrugCentral
chlorpromazine	Cytochrome P450 2D6	DrugCentral
chlorpromazine	Aldehyde oxidase	DrugCentral
chlorpromazine	FAD-linked sulfhydryl oxidase ALR	DrugCentral
nortriptyline	Cytochrome P450 2C19	DrugCentral

nortriptyline	Cytochrome P450 2D6	DrugCentral
nortriptyline	Aldehyde oxidase	DrugCentral
nortriptyline	Proto-oncogene tyrosine-protein kinase receptor Ret	DrugCentral
chlorpromazine	Calmodulin	DrugCentral
clozapine	GABA-A receptor*	Besnard et al. 2012, Squires and Saederup 1998[3,9]
chlorpromazine	GABA-A receptor*	Besnard et al. 2012[3]
nortriptyline	GABA-A receptor*	Squires and Saederup 1988[8]
imipramine	GABA-A receptor*	Besnard et al. 2012, Squires and Saederup 1988[3,8]
loxapine	GABA-A receptor*	Besnard et al 2012, Squires and Saederup 1998[3,9]
clotiapine	GABA-A receptor*	Squires and Saederup 1998[9]

All drugs and target full names, as well as their sources, as depicted in the chord diagrams in Supplementary Figure S13. All references are listed in the manuscript. Asterisk (*) refers to unspecified subtypes/isoforms.

Supplementary Table S10

Subtype	GABA [EC_{5-10}]	GABA [EC_{20-30}]	GABA [EC_{40}]	Source
α1β3	0.4μM & 0.5μM	1.5μM		Simeone et al 2017[12]
α5β3	0.2 μM	0.7μM		Varagic et al 2013[13]
α3β3	1μM	3μM		Varagic et al 2013[13]
α2β3	0.7μM	2.5μM & 3μM		Varagic et al 2013[13]
α1β3γ2	5μM			Ramerstorfer et al 2010[11]
**α1β3γ2 conc.**	15μM	50μM		Simeone et al 2019[14]
α1β2	1μM			Simeone et al 2017[12]
α1β2γ2	0.2μM	0.8μM		Varagic et al 2013[13]
α2β3γ1	3μM			Sieghart lab, unpublished
α2β3γ2	2.5μM			Ramerstorfer et al 2010[11]
α5β2γ2		2μM		Varagic et al 2013[13]
β2γ2		30μM	100μM	Wongsamitkul et al 2017[15]
α5β3γ2	1μM & 1.5μM	4μM, 4.5μM, 5μM		GABA DR in the manuscript
**α5F53W;L222Wβ3γ2**	1μM & 2μM	5μM & 6μM		GABA DR in the manuscript
**α5F53Wβ3γ2**	0.5μM & 0.8μM	1μM & 2μM		GABA DR in the manuscript
**α5L222Wβ3γ2**	0.8μM	1.5μM, 2μM, 3μM		GABA DR in the manuscript
**α5S189Wβ3γ2**	10μM	40μM		GABA DR in the manuscript
**α5L196Wβ3γ2**	20μM	50μM		GABA DR in the manuscript

GABA concentrations per subunit combination used for the various experiments in the manuscript, as well as the sources of GABA dose response curves. Since the EC_{20-30} is on the rising phase of the GABA dose response curve (for example, see Figure 4c), it amplifies variation that could occur from different mixtures of receptor populations expressed in an individual cell. In order to perform all experiments in the specified EC range, GABA concentrations were adjusted per cell within a narrow range as tabulated, and cells which still fell outside of this range were discarded.

References

1. Ashoor, A. et al. Effects of phenothiazine-class antipsychotics on the function of α7-nicotinic acetylcholine receptors. *European journal of pharmacology* **673**, 25-32 (2011).
2. Asproni, B. et al. Synthesis and pharmacological evaluation of 1-[(1,2-diphenyl-1H-4-imidazolyl)methyl]-4-phenylpiperazines with clozapine-like mixed activities at dopamine D(2), serotonin, and GABA(A) receptors. *J Med Chem* **45**, 4655-68 (2002).
3. Besnard, J. et al. Automated design of ligands to polypharmacological profiles. *Nature* **492**, 215-220 (2012).
4. Gotti, C., Riganti, L., Vailati, S. & Clementi, F. Brain neuronal nicotinic receptors as new targets for drug discovery. *Curr Pharm Des* **12**, 407-28 (2006).
5. Grinevich, V.P., Papke, R.L., Lippiello, P.M. & Bencherif, M. Atypical antipsychotics as noncompetitive inhibitors of alpha4beta2 and alpha7 neuronal nicotinic receptors. *Neuropharmacology* **57**, 183-191 (2009).
6. Korpi, E.R., Wong, G. & Luddens, H. Subtype specificity of gamma-aminobutyric acid type A receptor antagonism by clozapine. *Naunyn Schmiedebergs Arch Pharmacol* **352**, 365-73 (1995).
7. Singhal, S.K., Zhang, L., Morales, M. & Oz, M. Antipsychotic clozapine inhibits the function of alpha7-nicotinic acetylcholine receptors. *Neuropharmacology* **52**, 387-94 (2007).
8. Squires, R.F. & Saederup, E. Antidepressants and metabolites that block GABAA receptors coupled to 35S-t-butylbicyclophosphorothionate binding sites in rat brain. *Brain Res* **441**, 15-22 (1988).
9. Squires, R.F. & Saederup, E. Clozapine and Several Other Antipsychotic/Antidepressant Drugs Preferentially Block the Same ‘Core’ Fraction of GABAA Receptors. *Neurochemical Research* **23**, 1283-1290 (1998).
10. Weber, M.L. et al. Therapeutic doses of antidepressants are projected not to inhibit human α4β2 nicotinic acetylcholine receptors. *Neuropharmacology* **72**, 88-95 (2013).
11. Ramerstorfer, J., Furtmüller, R., Vogel, E., Huck, S. & Sieghart, W. The point mutation gamma 2F77I changes the potency and efficacy of benzodiazepine site ligands in different GABAA receptor subtypes. *Eur J Pharmacol* **636**, 18-27 (2010).
12. Simeone, X. et al. Molecular tools for GABAA receptors: High affinity ligands for β1-containing subtypes. *Scientific Reports* **7**, 5674 (2017).
13. Varagic, Z. et al. Subtype selectivity of alpha+beta- site ligands of GABAA receptors: identification of the first highly specific positive modulators at alpha6beta2/3gamma2 receptors. *Br J Pharmacol* **169**, 384-99 (2013).
14. Simeone, X. et al. Defined concatenated α6α1β3γ2 GABA(A) receptor constructs reveal dual action of pyrazoloquinolinone allosteric modulators. *Bioorg Med Chem* **27**, 3167-3178 (2019).
15. Wongsamitkul, N. et al. α subunits in GABA(A) receptors are dispensable for GABA and diazepam action. *Sci Rep* **7**, 15498 (2017).
